# Dynamic changes in neural circuitry during adolescence are associated with persistent attenuation of fear memories

**DOI:** 10.1038/ncomms11475

**Published:** 2016-05-24

**Authors:** Siobhan S. Pattwell, Conor Liston, Deqiang Jing, Ipe Ninan, Rui R. Yang, Jonathan Witztum, Mitchell H. Murdock, Iva Dincheva, Kevin G. Bath, B. J. Casey, Karl Deisseroth, Francis S. Lee

**Affiliations:** 1 Fred Hutchinson Cancer Research Center, Department of Human Biology, 1100 Fairview Ave N, Seattle, Washington 98109, USA; 2 Brain and Mind Research Institute, Weill Cornell Medical College, New York, New York 10065, USA; 3 Department of Psychiatry, Weill Cornell Medical College, New York, New York 10065, USA; 4 Sackler Institute for Developmental Psychobiology, Weill Cornell Medical College, New York, New York 10065, USA; 5 Department of Psychiatry, New York University School of Medicine, New York, New York 10016, USA; 6 Department of Cognitive, Linguistic, and Psychological Sciences, Brown University, Providence, Rhode Island 02912, USA; 7 Departments of Bioengineering and Psychiatry and Behavioral Sciences, Howard Hughes Medical Institute, Stanford University, Stanford, California 94305, USA

## Abstract

Fear can be highly adaptive in promoting survival, yet it can also be detrimental when it persists long after a threat has passed. Flexibility of the fear response may be most advantageous during adolescence when animals are prone to explore novel, potentially threatening environments. Two opposing adolescent fear-related behaviours—diminished extinction of cued fear and suppressed expression of contextual fear—may serve this purpose, but the neural basis underlying these changes is unknown. Using microprisms to image prefrontal cortical spine maturation across development, we identify dynamic BLA-hippocampal-mPFC circuit reorganization associated with these behavioural shifts. Exploiting this sensitive period of neural development, we modified existing behavioural interventions in an age-specific manner to attenuate adolescent fear memories persistently into adulthood. These findings identify novel strategies that leverage dynamic neurodevelopmental changes during adolescence with the potential to extinguish pathological fears implicated in anxiety and stress-related disorders.

## Abstract (web summary)

Flexible fear-related responses may be advantageous in adolescence. Here the authors use microprisms to image prefrontal cortical spine maturation across development and report that plasticity in adolescent fear extinction responses is associated with dynamic reorganization in the amygdalahippocampal-PFC circuit.

## Graphical abstract

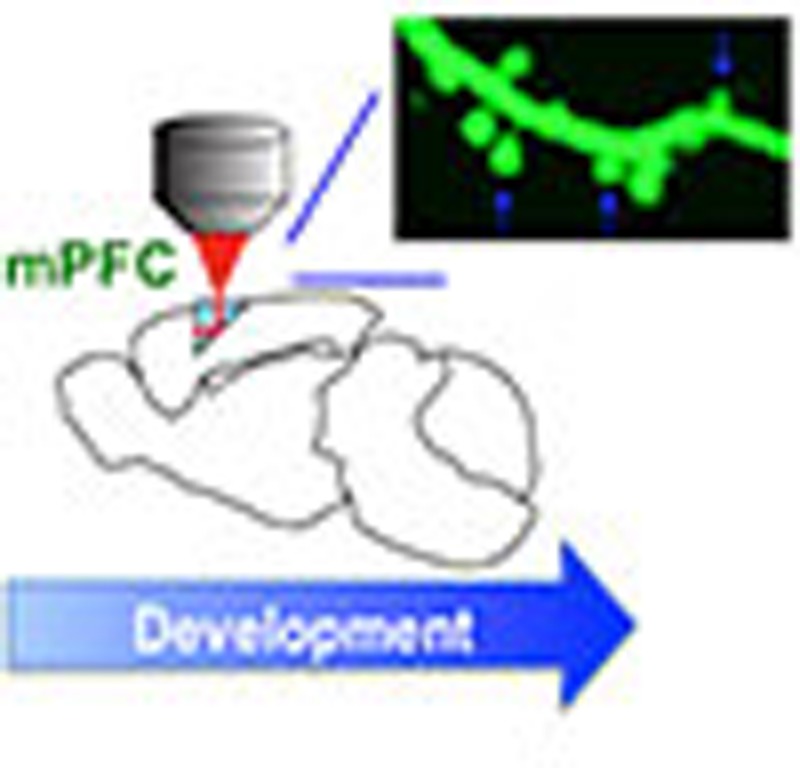

Fear learning emerges early in life^1^, but the capacity to express and extinguish fear memories undergoes dynamic changes across development. The capacity for extinction depends largely on whether the fear is specific to a cue or an environmental context. Specifically during adolescence, extinction learning for cued fear memory appears to be impaired^2^,^3^,^4^. During this same sensitive period, rodent studies have uncovered a coinciding sensitive period of suppressed expression of contextual fear^5^. These opposing fear-related behaviours depend on distinct components of a medial prefrontal cortical circuit (the infralimbic and prelimbic cortex) that receive projections from the amygdala and ventral hippocampus^6^,^7^. The infralimbic (IL) region of the medial prefrontal cortex (mPFC) has classically been recognized for its role in regulating fear expression via suppression of amygdala activity after extinction learning^6^, while the prelimbic (PL) region has been recognized for its role in sustaining responses during cue-related fear expression^8^. While this PL-amygdala input has long been known to be required for expression of a fear memory after conditioning, a novel role for the PL's involvement in mediating extinction has recently been identified. After extinction learning, ventral hippocampal inputs to PL play a critical role in gating the expression of fear memory by enhancing local inhibition and interfering with sustained PL activity^7^. Unlike neural circuits associated with fear to discrete cues, contextual fear requires the integration of spatial information in the environment via the hippocampus, through projections to both the amygdala and prefrontal cortex. Thus, both cued fear extinction and contextual fear expression rely on intact hippocampal projections to mPFC.

Developmental changes in the extinction of cued and expression of contextual fear memories may involve protracted development of prefrontal circuitry. Prefrontal circuits develop relatively slowly, undergoing a process of rapid synaptogenesis followed by accelerated synaptic pruning that extends into adolescence^9^,^10^,^11^,^12^. Here, we investigate whether the protracted development of PL and connectivity with ventral hippocampus and amygdala may be contributing to the disparate phenotypes observed in cued and contextual learning. To date, no studies have directly assessed the relationship between these behaviours and synapse formation in projections from hippocampus and amygdala to the mPFC, due in part to the inaccessibility of these structures to transcranial imaging techniques.

## Results

### Longitudinal imaging of spine dynamics in the prefrontal cortex

**Figure 1:**
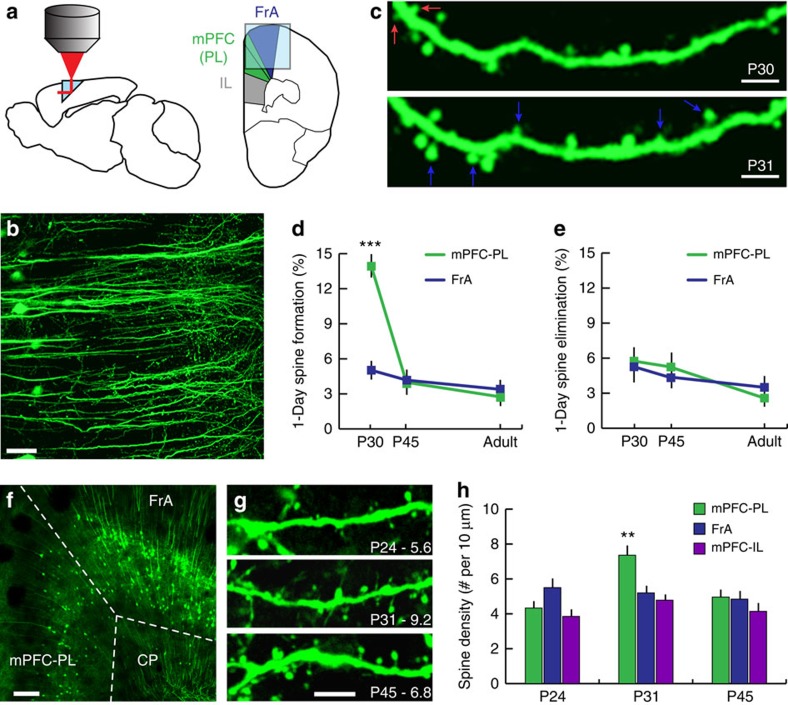
Imaging spine remodelling in mPFC. (**a**) Schematic for *in vivo* spine imaging in mPFC and FrA through a prism. The majority of analysed mPFC neurons reside in PL, although some may reside in the cingulate area of mPFC, dorsal to PL. IL neurons were excluded because they lie ventral to the prism. (**b**) YFP-expressing PL pyramidal neurons in Thy1/YFP-H line transgenic mice. (**c**) Spine remodelling was quantified in repeated images acquired at 1-day intervals. Eliminated spines (red arrow) were present at P30 but not P31. Formed spines (blue arrows) were present at P31 but not P30. (**d**) There were significant main effects of age (*F*(2,23)=87.3, *P<*0.0001) and cortical region (*F*(1,23)=41.4, *P<*0.0001) on spine formation (age X region interaction: *F*(2,23)=58.7, *P<*0.0001). Specifically, 1-day spine formation rates were selectively increased at P30 in mPFC compared with FrA (*P*<0.001, *post hoc* linear contrast). There were no significant regional differences at P45 or P90. (**e**) There was a significant main effect of age (*F*(2,23)=7.72, *P=*0.003) on spine elimination, but not of cortical region (*F*(1,23)=0.01, *P=*0.937). (**f**,**g**) Confocal images of YFP-expressing pyramidal cells in fixed tissue samples obtained at P24, P31 and P45. High-magnification images in **g** are from typical dendritic segments in PL (# spines/10 μm indicated at lower right). CP, Caudate/Putamen. (**h**) Quantification of developmental changes in spine density in PL, FrA and IL at P24, P31 and P45 (*N*=4–5 mice/age). There were significant main effects of region (*F*(2,31)=7.34, *P*=0.002) and age (*F*(2,31)=8.16, *P*=0.001), and an age X region interaction (*F*(4,31)=4.4, *P*=0.006), driven by an increase in spine density in PL at P31 relative to P24 (*P*=0.004) and P45 (*P*=0.007). Scale bars, 50 μm (**b**), 5 μm (**c** and **g**) and 150 μm (**f**). Error bars=s.e.m.; ***P<0.001; **P<0.01 for contrasts of P31 versus P24 and P45.

To study prefrontal synaptic plasticity and development across adolescence, we adapted a prism-based preparation that has been used in other cortical areas^13^,^14^ to allow for chronic two-photon imaging in the PL region of the dorsal mPFC (Fig. 1a), overcoming several technical obstacles that are unique to the objective of chronic imaging in this structure (Methods section). Briefly, after opening a small craniotomy over the midline, a custom borosilicate glass microprism (OptoSigma, Inc.) was stereotactically implanted in the dorsal mPFC in transgenic mice expressing yellow fluorescent protein (YFP-H line; Jax) in pyramidal cells of cortical layers 2/3 and 5 (Fig. 1a,b). The microprism was chronically fixed to the skull and the craniotomy was sealed using a combination of veterinary adhesives, to allow for repeated imaging in the developing adolescent brain.

We found that the implantation procedure was well tolerated: the mice were ambulating normally, eating and drinking, and grooming appropriately within 1–2 h after the surgery. Cortical lamination patterns and neuroanatomical structures adjacent to the prism were well preserved (Supplementary Fig. 1a). Consistent with prior reports^13^,^14^, prism implantation induced a modest but significant increase in cell density up to 50 μm from the prism face (Supplementary Fig. 1b,c). This was due in part to an increase in the density of astrocytes as detected by immunoreactivity for S100 calcium-binding protein B (S100B), a marker of mature glial cells (Supplementary Fig. 1b,d). There was no significant difference in cell density or astrocyte immunoreactivity at distances of 50–500 μm from the prism face, relative to a control area in the contralateral mPFC (Supplementary Fig. 1c,d).

After allowing 5–6 days of recovery, we used a customized two-photon laser scanning microscopy system to obtain high-resolution images of YFP-expressing pyramidal cells in the dorsal mPFC, capable of resolving postsynaptic dendritic spines (Fig. 1b,c). Using vascular landmarks and the contours of the microprism as a reference frame, we were able to return to precisely the same areas over protracted imaging intervals (Fig. 1c), enabling us to quantify the formation and pruning of postsynaptic dendritic spines on PL (but not IL; Fig. 1a) pyramidal cells in the developing dorsal mPFC. To test whether synaptic development in PL is associated with the decreased capacity for cued-fear extinction learning during adolescence^3^,^4^, we quantified formation and pruning rates for postsynaptic dendritic spines on the apical dendrites of pyramidal cells in the dorsal mPFC over a 24-h period from postnatal day (P)30 to P31, which is within the adolescent sensitive period. Because spine turnover rates were modestly but significantly altered at distances of <50 μm from the prism face (Supplementary Fig. 1e,f), corresponding to the area of increased astrocytic reaction, all subsequent analyses focused on dendritic segments >50 μm from the prism (50–200 μm). We found that 1-day spine formation rates (Fig. 1d) in the PL region of dorsal mPFC (mean±s.d.=13.9±1.6%) were nearly triple those observed in neighbouring frontal association (FrA) cortex (5.0±0.9%) at P30, but there were no differences in spine pruning (5.8±1.4% in the PL region of dorsal mPFC versus 5.4±1.6% in FrA) during this period (Fig. 1e). To control for the possibility that these effects were caused by severing some long-range projections to mPFC, we used a recently published protocol^15^ to image mPFC across the midline through a prism implanted in the contralateral hemisphere (Supplementary Fig. 2a,b), preserving the integrity of ipsilateral circuits. Spine formation (12.5±1.6%) and elimination rates (5.3±1.3%) were statistically indistinguishable in this preparation (Supplementary Fig. 2c; *P*>0.38, Mann–Whitney). Furthermore, when the same experiment was performed in separate cohorts of mice during late adolescence (prism implantation at ∼P39, imaging at ∼P44–P45) and young adulthood (prism implantation at ∼P84, imaging at ∼P90–P91), there were no significant differences in 1-day spine formation or pruning rates between the two regions (Fig. 1d,e). Together, these data demonstrate that the onset of a developmental period of impaired fear extinction coincides with a surge in formation of excitatory postsynaptic dendritic spines that occurs specifically in mPFC.

Next, we tested whether this selective increase in spine formation, but not elimination, was associated with regionally selective, developmental changes in spine density in fixed tissue samples obtained from YFP-H line transgenic mice before, during and after this developmental surge in spine formation (that is, at P24, P31 and P45; Fig. 1f,g). Furthermore, we also characterized developmental changes in spine density in the IL region of ventral mPFC, which is inaccessible through a 1.5-mm prism. In accord with the effects we observed on spine dynamics *in vivo*, we found that the surge in spine formation in the PL region of dorsal mPFC was associated with a significant increase in spine density at P31 (7.4±1.3%), compared with P24 (4.3±0.7%) and P45 (5.0±0.8%; Fig. 1h). In contrast, spine density tended to decrease gradually with age in FrA and IL (Fig. 1h). Together, these experiments indicate that the rapid decline in extinction learning at P30 coincides with a surge in spine formation and spine density selectively on the apical dendrites of pyramidal cells residing in cortical layers 2/3 and 5 in the PL region of dorsal mPFC, but not in IL or FrA. These findings are consistent with our previous findings of enhanced excitatory synaptic transmission in layer 5 pyramidal neurons in the PL of P30 mice, compared with younger and older mice^4^.

### Retrograde tracer analysis of dynamic neural connectivity

**Figure 2:**
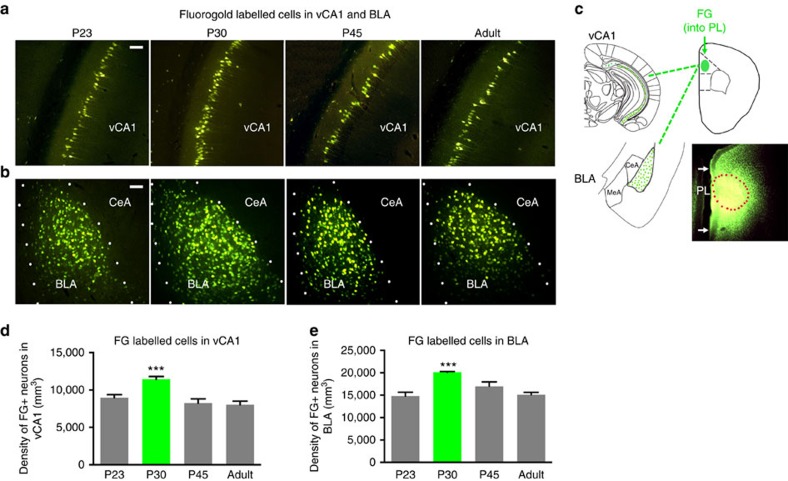
Dynamic developmental surge in neural connectivity to mPFC. Representative images of FG-labelled neurons in (**a**) hippocampus ventral CA1 (vCA1) and (**b**) basolateral amygdala (BLA), Scale bars, 50 μm. (**c**) Schematic of FG injection in prelimbic cortex (PL) and FG+ cells throughout vCA1 and BLA. Outlined area denotes injection site, with surrounding diffusion maintained within PL boundaries. Quantification of density of FG (FG+) retrograde labelled neurons in showed a significant main effect of age in (**d**) vCA1, *P*<0.0005, *F*(3,16)=11.58, vCA1 (P23 8970.7±401.3; P30 11441.1±368.8; P45 8254.4±565.1; P90 8024.6±479.5) and a significant main effect of age in (**e**) BLA, *P* <0.0005, *F*(3, 16)=12.2, BLA (P23 14809.1±847.3; P30 20124.1±165.6; P45 16995.1±973.0; P90 15114.8±520.5).

As these apical dendrites are the primary recipients of long-range projections from other cortical and subcortical areas, we hypothesized that developmental changes in projections from the ventral hippocampus and amygdala may contribute to changes in spine formation and diminished extinction learning at P30 (ref. 16). To test this hypothesis, a retrograde tracer, Fluorogold (FG), was stereotactically injected into the PL of P23, P30, P45 and adult (P60) mice to directly assess hippocampal and amygdala projections to the PL (Fig. 2). Visualization and quantification of FG-labelled cell bodies via immunohistochemistry revealed dense populations of PL-projecting cell bodies within the BLA (Fig. 2b) that increased significantly from P23 to P30, and subsequently decreased by P45 (Supplementary Figs 3–5 and Supplementary Tables 1,2) for injection site, topographic features and control regions. This transient increase in FG labelling in the BLA, may be the consequence of increased arborization of BLA inputs to PL or increased density of BLA neurons projecting to PL, and as a consequence could result in an increase in AMG influence on PL activity. These findings indicate that neurodevelopmental increases in structural connectivity in the BLA-PL circuit may contribute to diminished extinction learning previously observed during early adolescence, as BLA input to PL may activate a positive feedback loop for maintaining fear expression^7^,^17^. Subsequent synaptic pruning of the PL-BLA circuit during the transition from adolescence to adulthood may contribute to the re-emergence of intact extinction learning in both rodents and humans^4^, as this PL-BLA circuit involves a highly characterized positive feedback loop implicated in fear expression and undergoes increased structural connectivity only during adolescence when cued fear is resistant to extinction.

Interestingly, these retrograde labelling experiments also revealed a significant surge in connectivity between ventral CA1 (vCA1) and PL that peaked at P30 and decreased by P45 (Fig. 2a,c,d and Supplementary Figs 3 and 4)—the same developmental period during which contextual fear expression is suppressed. FG labelling in the claustrum, thalamus, IL and other areas of hippocampus revealed no such developmental changes in PL connectivity from P23 to adulthood (Supplementary Fig. 5). In addition, FG was stereotactically injected into the IL at the same ages and hippocampal and amygdala projections to the IL assessed. Unlike the PL, there was no significant increase in vCA1 or BLA projections to the IL from P23 to P30 or P45 (Supplementary Fig. 6), suggesting further the selectivity of the increase in region-specific projections to the PL across this developmental period. Previous studies have shown that ventral hippocampal inputs serve to dampen the sustained, fear-related activity in PL, and may serve a gating function for fear-enhancing inputs from the amygdala^7^ particularly in the setting of ambiguous contextual information^18^. Thus, the selectively enhanced inputs from BLA and ventral hippocampal neurons to the PL provide structural correlates for the surge in spine formation that occurs at P30 (Fig. 1d). While increased BLA inputs to PL correlate with sustained extinction-resistant cued fear, increased ventral hippocampal inputs to PL may contribute to fearlessness for contextual elements observed during the same sensitive period^3^,^4^,^5^.

### Leveraging connectivity findings to optimize fear extinction

**Figure 3:**
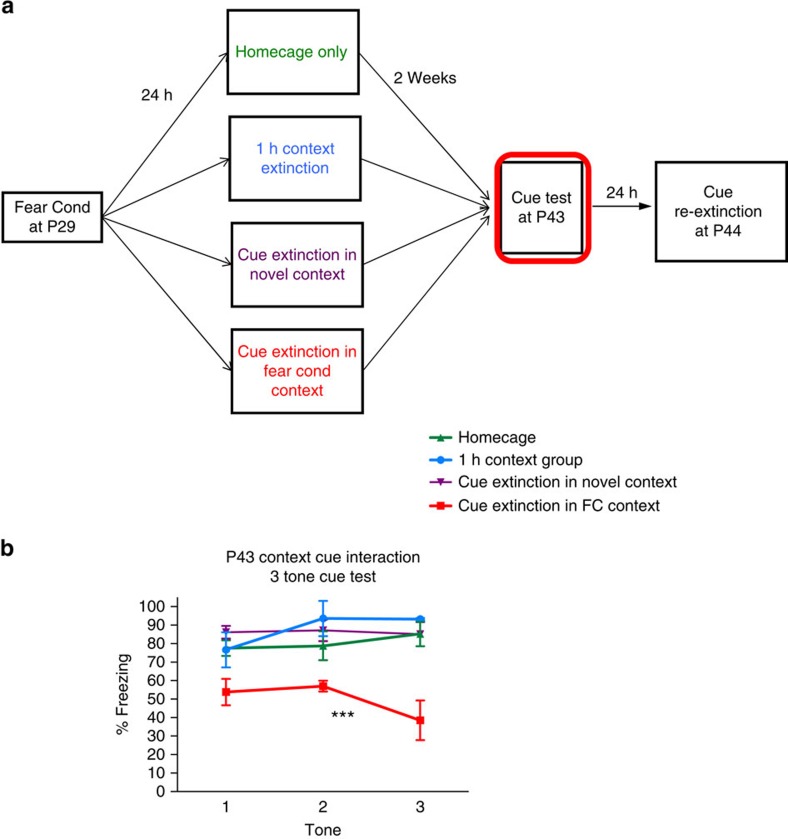
Combined contextual and cue extinction effects on long-term memory. (**a**) Behavioural paradigm depicting four groups designed to assess if there is an interaction between cued fear extinction and the context in which it is presented for mice fear conditioned at P29 and tested during late adolescence (P44). (**b**) Three tone test at P44 for all four behavioural treatment groups demonstrates significantly less freezing in the group that underwent cued fear extinction in the fear-conditioning context (*F*(3,12)=14.7, *P*<0.0005).

Given that hippocampal inputs to PL are capable of suppressing fear expression^7^, might activation of this circuitry during adolescence via contextual elements ameliorate the diminished cued-fear extinction observed during this developmental window (that is, enhance extinction via hippocampal-mediated inhibition of PL neurons)? To this end, we designed a behavioural intervention that maximally targeted the contextual component of a prior conditioned cued fear^19^,^20^. While the degree of persistent attenuation of contextual fear in adults was restricted to previously described retrieval-based time windows, persistent attenuation of contextual memories in adolescents showed enhanced flexibility that correlated with a lack of synaptic potentiation in basal amygdala (Supplementary Figs 7–10; Supplementary Notes 1 and 2). Because adolescent mice show enhanced capacity for contextual extinction, but also a robust cued fear that is highly resistant to cued extinction (Supplementary Figs 11 and 12), we tested whether we could improve extinction to discrete cues by bootstrapping onto the enhanced capacity for contextual extinction during this time. Mice were conditioned at P29 and then remained in their home cages or were subjected the following day to either a contextual extinction session, a cued extinction session in a novel context, or a cued extinction session in the original fear conditioning context at P30 (Fig. 3a). Mice were then tested for freezing to the tone in a novel context 2 weeks post-conditioning and subsequently given a re-extinction session to see if a previously acquired discrete cued fear could be attenuated during adulthood. Interestingly, mice that were cue-fear conditioned during adolescence and then left in their home cage without further manipulation maintained a heightened freezing response in adulthood that was resistant to subsequent extinction (Supplementary Fig. 13), highlighting the importance of the age at which the fear was acquired. The cohort that received cued fear extinction in a novel context and the cohort that received context-only extinction also maintained a heightened robust fear response during adulthood, likely due to the aforementioned lack of successful extinction during adolescence. The cohort that received a combination of both contextual and cued fear extinction exhibited a significant decrease in freezing to tone cues and was the only cohort that successfully re-extinguished. These findings suggest that a combination of context and cued fear extinction may offer benefits during this sensitive window of development (Fig. 3a,b, Supplementary Fig. 13). While the combined context and cue extinction sessions did offer a benefit over cued-only or context-only extinction for all age groups, including adults, the effect is particularly interesting for the adolescent cohort, as this coincides with a time when their cued-only extinction is insufficient to significantly decrease fear memory (Fig. 3 and Supplementary Figs 11–14 and Supplementary Notes 1 and 2).

Fear renewal, or the return of fear on experiencing a reminder cue outside of the extinction context, remains a major obstacle for clinical treatment of anxiety disorders in humans^21^,^22^ and may be the result of tipping the balance between activation-specific neuronal circuits in the hippocampus and amygdala^23^. This clinical observation highlights the need for better treatment methods, perhaps through further investigation of hippocampally mediated techniques, such as contextual extinction combined with discrete cue reminders. The experiments presented here provide a more detailed framework for when, developmentally, contextual-based techniques can be most beneficial.

## Discussion

In summary, we have identified discrete and selective surges in spine formation and increased spine density, within the PL area of dorsal mPFC, as well as enhanced structural connectivity between PL and vCA, and PL and BLA during a period in adolescence in which we observe two disparate effects on fear behaviours (heightened cued fear that is resistant to cued fear extinction and contextual fear suppression). We leveraged a new preparation for imaging mPFC through chronically implanted microprisms to identify these developmental changes. Previous studies from human and nonhuman primates have shown that the adolescent period is associated with overproduction, followed by selective stabilization and elimination, of principally excitatory synapses in the cortex^9^,^10^. Our anatomical findings highlight a selective and dynamic reorganization of synaptic spine circuitry within the PL, but not in the IL, during this period. In addition, as compared with other cortical regions, the PFC has been shown to have one of the highest levels overproduction of spines across postnatal developmental and it has previously been shown that during adolescence, the subsequent pruning of spines within different PFC layers (IIIc, V) occurs at different rates^24^,^25^,^26^.

The timing of elevated production of synaptic spines in PL coincides with a period of resistance to cue extinction learning and enhanced capacity for contextual fear attenuation. Curiously, during this same period we observed significantly diminished expression of contextual fear. As recent work has demonstrated that projections from BLA to PL enhance fear expression via activation of excitatory pyramidal cells within PL (ref. 7), our observed selective surge in BLA-PL connectivity could support a cyclic loop of BLA-PL and PL-BLA activity during adolescence that correlates with resistance to cued fear extinction at this age. Previous studies using anterograde tract-tracing analyses have demonstrated a significant increase in BLA connectivity to the PFC during adolescence^11^. Our observation of enhanced BLA-PL at P30 is consistent with these previous findings. However, BLA inputs into the IL do not appear to undergo a similar surge in connectivity at this P30 time point (Supplementary Fig. 6), highlighting the potential for selective activation of the BLA-PL circuit during adolescence. The underlying molecular mechanism that may drive a selective enhancement of connectivity to between the BLA and PL remain to be determined. Multiple growth factors and neuromodulators have been shown to increase during this developmental window in cortex, hippocampus and amygdala, including brain-derived neurotrophic factor^27^. However, we are not aware of any reports indicating that any of these factors might be modulated in a regionally selective manner to support the preferential enhancement of inputs to the PL and further studies are warranted.

While the surge in connectivity between vCA1 and PL may be insufficient to override the PL-BLA circuit during cue extinction, enhanced vCA1-PL circuit activation during contextual extinction may persistently attenuate the contextual fear (Supplementary Fig. 8), likely due to vCA1 projections to inhibitory GABAergic neurons^7^ within PL. Utilizing the enhanced capacity for contextual fear attenuation during adolescence, we showed that contextual fear can be persistently attenuated with a single extinction session during this adolescent period. However, once outside this ‘sensitive period' between P29 and P43, this form of extinction has minimal effects in reducing contextual fears acquired in adulthood. Finally, exploiting this capacity for contextual extinction with a combined context-cue extinction session offers a significant benefit to cued fear extinction in the adolescent time frame.

These findings also demonstrate that fear memories can be blocked and persistently suppressed without ever being expressed during adolescence in mice. This unexpected discovery implies that prophylactic behavioural treatments aimed at preventing the emergence of anxiety-like symptoms after traumatic or stressful experience through contextual means may prove more beneficial than waiting for symptoms to emerge before intervening. Previous studies have shown that acute or chronic stress in adolescence leads to long-lasting anatomical and behavioural consequences, especially with regard to anxiety and fear-related behaviours^28^,^29^,^30^. Our findings provide one of the first indications for developmentally informed treatments for preventing the emergence of anxiety disorders following stressful or traumatic events, even if they are asymptomatic, as a form of prophylactic treatment. These findings also highlight the potential importance of a contextual extinction-based therapy and preventive interventions for youth. Such non-invasive behavioural interventions, as part of resilience training, may help to inoculate against fear memories and improve future outcomes in children and adolescents exposed to stressful or traumatic events, warranting the need for future studies to explore the mechanistic link between the changes in prefrontal cortical development and contextual fear learning discussed here.

## Methods

### Surgical implantation of an intracranial microprism

Custom glass microprisms (OptoSigma, Santa Ana, CA) were intracranially implanted in transgenic mice expressing YFP in layers 2/3 and 5 pyramidal cells (Thy1/YFP-H line, Jackson Labs, Bar Harbor, ME). The microprisms used in this study were square, right-angle prisms, 1.5 mm on a side and made of (BK7) borosilicate glass with a reflective aluminium coating on the hypotenuse. Because the aluminium coating was not always stable over long-imaging intervals, an additional silicon dioxide protective coating was applied on the external surface of the hypotenuse.

The implantation procedure was performed 5–6 days before imaging. Thus, separate cohorts of mice were used for each developmental time point, for example, in one cohort, the microprism was implanted on ∼P24 for imaging at P30–P31, and in a second cohort, the microprism was implanted on ∼P39 for imaging at P44–P45. Buprenorphine (0.5 mg kg^−1^ IP) and dexamethasone (1 mg kg^−1^ IP) were administered before surgery for prophylactic analgesia and to reduce perioperative inflammation, respectively. Surgical-plane anaesthesia was achieved using isoflurane (5% for induction, 2–3% for maintenance), monitoring for depth of anaesthesia every 10 min throughout the procedure. A sterile eye lubricant was applied to both eyes to prevent corneal drying, and normothermia was maintained using a heating pad. After removing the fur on the scalp using a scissors, the animal's head was stabilized in a stereotaxic device using ear bars and a nose restrainer (David Kopf Instruments, Tujunga, CA). The scalp was then prepped for surgery with a Betadine scrub.

Next, the skin superficial to the skull was incised medially and reflected away using sterile surgical instruments, and a small circular section of skin (∼0.75 cm in diameter) was excised. The periosteum was bluntly dissected away. Using the digital stereotaxic device, the skull overlying the prefrontal cortical area to be imaged was located in stereotaxic coordinates (0–1.5 mm lateral to midline; ∼1.7–1.8 mm anterior to Bregma). The precise location of the area to be imaged along the anterior-posterior axis was selected to avoid transecting large blood vessels, which could be visualized by thinning the skull in phosphate buffered saline using a compact dental drill and sterile drill bit. A circular head plate was then centred over the area of interest and fixed to the skull using Metabond dental cement (C&B Metabond, www.parkell.com). Next, a rectangular craniotomy slightly larger than the prism (∼1.7 × 1.7 mm, flush with the midline and the prefrontal area of interest) was opened using a dental drill. To facilitate insertion of the prism into the brain, a small incision (∼1.6 mm) was made in the dura using a fresh sterile scalpel blade, and the dura beneath the craniotomy was gently dissected away, taking care not to compress the underlying brain tissue or puncture the sagittal venous sinus. This resulted in no more than trace bleeding that could be wiped away using a sterile Sugi cellulose spear (John Weiss & Son, Ltd). Throughout the procedure, the area was irrigated regularly with artificial cerebrospinal fluid. At this stage in the procedure, the area was ready for implantation of the microprism.

Relative to other cortical areas, we found that the prefrontal cortex was more sensitive to damage due to compression during the insertion process and more prone to bleeding due to the presence of the midline venous sinus and large blood vessels emanating from this structure. To mitigate the former problem, we used a digital stereotaxic micromanipulator (David Kopf Instruments) to aid in the insertion process. The microprism was attached to a syringe with a 25G, blunt-tipped needle using a central vacuum line. The syringe, in turn, was attached to a standard electrode manipulator and digital display console (Kopf Model 940) to achieve micrometre-precision manipulation of the position of the microprism. In this way, the microprism was lowered slowly into the brain over the course of ∼5 min in increments of ∼100 μm, allowing time for the tissue to adjust and accommodate the volume of the prism. This process was continued until the upper surface of the prism was nearly flush with the dorsal surface of the cortex. We also took two measures to reduce the risk of bleeding during the insertion process. First, the face of the prism was positioned such that it did not bisect major vasculature to the extent this was possible. Second, the prism was initially inserted ∼100 μm lateral to midline to avoid contact with the midline venous sinus during the insertion process. After lowering the prism to the desired location (flush with the dorsal surface of the cortex), the prism was gently moved medially using the micromanipulator until it was flush with the midline. Using these measures, intraoperative bleeding was minimal. When the microprism was successfully positioned in the desired location, the central vacuum line was turned off, allowing the blunt needle to disengage from the surface of the prism without disturbing it or the adjacent cortex.

After inserting the prism and disengaging the syringe needle, we applied a thin layer of 1% agarose to the surface of the brain along the perimeter of the implant to protect the underlying tissue. Next, a veterinary adhesive (Vetbond, 3M Inc., St. Paul, MN) was applied to adhere the prism to the skull. Finally, a layer of Metabond was applied for added durability in the fixation of the prism to the skull. At the end of the procedure, this layer of Metabond adhered to the prism, skull and head plate, and covered all areas of exposed skull. We found that this preparation was typically highly stable over imaging intervals of many months.

To control for the possibility that spine turnover rates may be altered by severing long-range projections to and from mPFC on implantation of the microprism, we used a preparation inspired by a recently published protocol for imaging mPFC across the midline through a microprism implanted in the contralateral hemisphere^15^. By implanting in the contralateral hemisphere, this preparation spares ipsilateral projections to and from mPFC. Here, the prism was rotated 90° and implanted in the contralateral hemisphere, with the face in a sagittal plane instead of a coronal plane (Supplementary Fig. 2A). In all other respects, the procedure was identical to that described above. The results (*N*=3 mice) are reported in Supplementary Fig. 2.

### Two-photon imaging and analysis of spine dynamics

All images were acquired using a custom two-photon laser-scanning microscope (Prairie Technologies, Middleton, WI) equipped with a scanning galvanometer and a Spectra-Physics Mai Tai Ti:Sapphire laser (Spectra-Physics, Santa Clara, CA) tuned to 920 nm. All images were acquired through a × 25, 1.05 numerical aperture objective with a 2-mm working distance (Olympus XLPlan N, Olympus America Inc., Center Valley, PA) and a correction collar partially correcting for optical aberrations due to the prism. In each imaging session, Z stacks (typically 512 × 512 pixels, 4–8 μs pixel dwell time, 1-μm steps, 1–1.5 × optical zoom) were acquired through the microprism at distances of 5–250 μm from the prism face. We used stable vascular landmarks and the contours of the prism to relocate the same neurons and dendritic segments with micrometre precision across imaging sessions. Spine turnover during a given imaging interval was analysed using the freely available ImageJ software package (imagej.nih.gov) to compare pairs of images. Eliminated spines (present in image 1, absent in image 2) and formed spines (absent in image 1, present in image 2) were quantified as a per cent of the total number of spines identified in the initial image, using criteria defined in previously published work^31^,^32^,^33^. As in those previous works, thin, filopodia-like structures (length>3X width at the widest point) were excluded from analysis. We found that spine elimination was modestly increased, and spine formation was modestly decreased, at distances of 0–50 μm from the prism face compared with distances of 50–100 μm, 100–150 μm and 150–200 μm (Supplementary Fig. 1e,f). Image quality tended to degrade at distances>200 μm from the prism face, making it difficult to resolve individual spines. Furthermore, it was difficult to resolve individual spines at distances of <75 μm from the cortical surface, due to the increased density of YFP expression in this area of intense dendritic branching and arborization. Therefore, in both mPFC and FrA, all subsequent analyses of spine turnover, including all analyses reported in the main text, were restricted to dendritic segments located 50–200 μm from the prism face, or ∼1.8–2.0 mm anterior to Bregma and 75–300 μm from the pial surface. This area overlapped with the area of PL that was the subject of the tract-tracing studies described below. To test for regionally specific, developmental changes in spine formation and elimination, we used a two-factor (age X region) analysis of variance (ANOVA) and *post hoc* linear contrasts. Main effects of age and region and the interaction between these two factors are reported in Fig. 1.

As an additional control, we also tested whether 1-day spine elimination and formation rates varied as a function of distance from the prism face, using Kruskal–Wallis ANOVA and *post hoc* contrasts (Dunn's Test) to identify specific between-group differences. As reported in Supplementary Fig. 1e–f, we found that spine elimination was modestly but significantly increased, and spine formation was decreased, at distances of 0–50 μm from the prism face, compared with greater distances from the prism face. Therefore, all analyses reported in the main text were for spines located >50 μm from the face of the prism. Finally, to control for the possibility that spine remodelling was altered by severing long-range projections to mPFC on implantation of the prism, we used a recently published protocol to image mPFC across the midline through a prism implanted in the contralateral hemisphere, preserving the integrity of ipsilateral projections, and we used Mann–Whitney *U* tests to compare spine formation and elimination rates in the mPFC in these two preparations. The results are reported in Supplementary Fig. 2.

### Confocal imaging and analysis of spine density and histology

Motivated by the observation that spine formation but not elimination was selectively increased in PL but not FrA from P30 to P31, coinciding with a surge in projections from vCA1 and BLA to PL but not IL, we tested for corresponding changes in spine density. YFP-H line transgenic mice were transcardially perfused with a paraformaldehyde solution at P24, P31 and P45, and 100-μm-thick coronal sections were prepared and mounted on slides for imaging. Images were acquired on a laser scanning confocal microscope (Olympus Fluoview) using a 440-nm laser, a 60X, 1.30 NA oil-immersion objective. 50–80 μm image stacks were acquired at 512 × 512 pixel resolution, 3X digital zoom, 8–10 μs pixel dwell time and 0.75 μm distance between images. Spine density was quantified by counting spines per unit length on randomly selected segments of apical dendrites in sections located 1.7–2.2 mm anterior to Bregma at distances of 75–300 μm from the cortical surface (chosen to coincide with the locations analysed in the *in vivo* imaging experiments), selected using the same criteria defined for the in vivo imaging experiments (and excluding filopodia, as above). Because the density of cells expressing YFP increases over time in this transgenic line (especially in the IL region of mPFC), dendritic segments were included in this analysis only for cells exhibiting robust YFP expression throughout the apical dendrite, extending from the soma toward the cortical surface. Four to five mice were used for each time point (P24, P31 and P45). We found that our estimate of spine density stabilized after counting 300–400 spines per region; therefore, we conservatively counted ∼500 spines per region per mouse to obtain the spine density estimates depicted in Fig. 1. As above, we used a two-factor (age X region) ANOVA and *post hoc* linear contrasts to test for effects of age and cortical region on spine density. The results are reported in Fig. 1h.

In addition, we tested for changes in cell density and gliosis as a function of distance from the prism face in a separate cohort of mice (*N*=5). As described below, the mice were transcardially perfused with a paraformaldehyde solution ∼1 week after prism implantation at ∼P24 (corresponding to the recovery period used in the *in vivo* imaging experiments), and 40-μm sections were prepared and mounted on slides after staining for DAPI and S100B, a marker of mature glial cells. Thirty-micrometre image stacks were acquired using a 408-nm laser (for DAPI) and a 559-nm laser (for S100B), a × 40 oil-immersion objective, and no digital zoom; imaging parameters were otherwise identical to those used above. Cell densities (DAPI+) and glial cell densities (S100B+) were quantified by counting cells per unit area in zones defined in 25-μm increments from the prism face at distances of up to 500 μm (100 μm increments were used for distances >300 μm). Cell densities were assessed with respect to a corresponding area located in approximately the same place in the contralateral hemisphere, and are reported in Supplementary Fig. 1. For both analyses, Kruskal–Wallis ANOVA and *post hoc* contrasts (Dunn's Test) were used to test for an effect of distance from the prism face on cell density (DAPI+) and gliosis (S100b+). The results are reported in Supplementary Fig. 1c–d.

### Immunofluorescent histology

All experiments were carried out at room temperature unless otherwise specified. After two-photon imaging, the mice were killed by intraperitoneal injection of Sleep-Away and perfused transcardially as described in the retrograde tracing section. Coronal and sagittal sections (40 μm) of whole brain were cut by using a sliding microtome frozen by powdered dry ice. Free-floating serial sections (taken one every third) were washed in TBS, incubated for 30 min in a blocking solution containing 4% normal horse serum (vol/vol), 1% BSA in TBS with 0.2% Triton X-100 (TBS-Tx) and incubated overnight at 4 °C with rabbit anti S100b primary antibody (HPA015768, Sigma) diluted 1:500 in the blocking solution mentioned above. Sections were then washed in TBS and incubated for 2 h with Alexa Fluor-labelled donkey anti-rabbit IgG secondary antibody (Alexa Fluor -555) diluted 1:500 in TBS-Tx. After washing three times for 10 min, sections were mounted onto chrom-alum/gelatin-coated slides, and air-dried for 2 h in the dark. Slides were cover-slipped by water-soluble glycerol-based mounting medium containing DAPI, and sealed with nail polish. As described above, Kruskal–Wallis ANOVA and *post hoc* contrasts (Dunn's Test) were used to test for an effect of distance from the prism face on cell density (DAPI+) and gliosis (S100b+). The results are reported in Supplementary Fig. 1c,d.

### Retrograde tracing and cell counting

Individual C57BL/6J mice of specified age groups (Charles River, Wilmington, MA) were microinjected stereotactically with the retrograde fluorescent tracer, FG (Fluorochrome, Denver, CO). Anteroposterior, mediolateral, dorsoventral coordinates for PL and IL are listed in Supplementary Table 1 and Supplementary Table 2. FG was dissolved in neutral saline to yield a 4% concentration. Mice were anaesthetized with a cocktail of ketamine and xylazine (100 mg and 10 mg ml^−1^) at dosages of 0.1 ml per 10 g body weight, then mounted on a stereotaxic frame (David Kopf Instruments, Tujunga, California USA). After surgical exposure of skull, a 3% of H_2_O_2_ solution was applied on the top of skull to enhance skull sutures and bregma. Stereotaxic coordinates were performed by using an electrical drill mounted on a manipulator (David Kopf Instruments, Tujunga, California) and micro-injection was performed with a Nanoject II (Drummond Scientific Nanoject II, Fisher Scientific, USA) attached to another manipulator. Glass micropipettes for injection were made with outside tip diameters of 25 μm and a total volume of 10 nl of 4% FG was injected three times at an interval of 2 min for each mouse. The injection needle was kept at place for 10 min and withdrawn slowly. After 7 days, mice were deeply anaesthetized with sodium pentobarbital and perfused transcardially with 30 ml of saline wash followed by 120 ml of fixative (4% paraformaldehyde in 0.01 M sodium phosphate buffer, pH 7.4) by using Perfusion Two automated pressure perfusion system (Leica Microsystems, Buffalo Grove, IL, USA) at a flow rate of 25 ml min^−1^. For FG injections, animals were injected with a fixed volume of FG (0.01 μl per animal). Given the combination of the small volume being injected and previous work demonstrating minimal changes in brain volume and fluid content over this abbreviated period of development^34^, we did not change the injection volume to account for differences in somatic weight changes. In addition, as this is a retrograde tracer and minimal amounts are required for neuronal uptake, we chose not to adjust based on volume of injection area relative to total volume of PL. The brains were then removed and post-fixed with the same fixative at 4 °C overnight and transferred to a sucrose solution (30% sucrose in 0.1 M phosphate buffer) at 4 °C for 48 h. Coronal sections (40 μm) were cut on a freezing microtome. One section of every three was mounted in the correct order and direction. The other sections (between every third) were collected in an antifreeze solution (30% Glycerol, 30% ethylene glycol and 40% 0.25 M phosphate buffer) and stored at −20 °C, such that three total sets of series representative sections were taken and for each set sections were 120 μm apart. The mounted set slides were air dried for 3 h and cover-slipped by using a novel mounting solution (containing 10% SiO_2_, 0.1 M Tris, pH=11). The use of 10% SiO_2_ in high pH in the mounting solution enhanced the fluorescence of FG significantly, thus avoiding the need for standard immunohistochemistry to visualize FG labelling. This method also allowed for more accurate quantification of data by avoiding issues often associated with immunohistochemistry, such as variable staining due to uneven penetration of IgG (15 μm). Previous immunohistochemistry techniques often use 40 μm tissue sections, but usually leave 10 μm in the middle un-stained such that the section forms 'sandwich-like' staining pattern that can interfere with the accuracy of quantification. Since there is no need for secondary antibody staining of floating sections using this method that allows for direct measurement of FG fluorescence, sections could be mounted immediately after slicing, which allowed for intact serial sections avoiding any section loss, and facilitating systemic sampling and stereological analysis. Serial sections were imaged with a Nikon 80i fluorescent microscope with a DAPI/FITC/Rhodamine Tri-color Filter. Microphotographs were acquired using a MicroFire digital camera and FireFrame software (Optronics, Princeton, NJ, USA) and stereological estimation of cell density was performed using StereoInvestigator Software (MicroBrightfeild, VA, USA). Stereological estimation of area of injection sites and retro-labelled cell density in brain regions strictly followed stereological roles. Briefly, systemic random sampling by choosing one of three sections that includes PL as starting sections, then take sections at same interval of 120 μm until ventral hippocampus disappears. By referring to Allen Brain Atlas, 6–7 regions were traced with a 4x lens, then stereological estimation was performed by using different probes. Injection sites were screened and judged, and only mice with injections mainly limited to the rostral 2/3 of PL and well limited in IL were included. Then injection site coverage was estimated by using Cavalieri estimator and calculated by divided volume of injection site by total PL volume. Contours of basolateral nucleus (BLA) and ventral hippocampus, claustrum and medial dorsal thalamic nucleus were made, and systemic randomly sampling grid was applied to drawn contours. Total volume of brain regions were estimated by using Cavalieri estimator and total cell numbers were estimated by Fractionator with counting frame size 25 × 25 × 40 μm and sampling grid size 100 × 100 μm. We used a one-way ANOVA to test for main effects of age for each region along with *post hoc* analyses (Tukey) to test for differences between select groups.

### Electrophysiology

Brains were quickly removed after pentobarbital anaesthesia and placed in ice-cold artificial cerebrospinal fluid of the following composition (in mM): NaCl (118), KCl (2.5), glucose (10), NaH2PO4 (1), CaCl2 (1), MgSO4 (2) and NaHCO3 (25) bubbled with 95% O2/5% CO_2_ (pH 7.4). Coronal slices (400 μm) containing amygdala were cut with a vibratome and maintained at room temperature for 90 min in a brain-slice keeper to allow recovery. A single slice was then transferred to a recording chamber perfused with artificial cerebrospinal fluid as described above but containing 2 mM CaCl_2_. The temperature of the chamber was maintained at 32 °C with a TC324B in-line solution heater and controller (Warner Instruments). The basal amygdala principal neurons were visualized using video-enhanced infrared differential interference contrast microscopy (Hamamatsu C5405), with an Olympus BX50WI upright microscope fitted with a × 40-long working distance water-immersion objective. Patch electrodes (4–6 MΩ) were filled with an intracellular pipette solution consisting of the following (in mM): CsCl (130), HEPES (10), EGTA (0.5), QX-314 (5), GTP (0.2) and MgATP (5). Osmolarity was adjusted to 290 mOsm with sucrose, and pH was adjusted to 7.4 with CsOH. Excitatory postsynaptic currents (EPSCs) were recorded at −60 mV in the basal amygdala neurons by electrical stimulation of the perirhinal cortex using an extracellular electrode as described before in the presence of bicuculline (10 μM)^5^. Spontaneous EPSCs (sEPSCs) were recorded at −60 mV, in the presence of bicuculline. Recordings were made using an Axopatch 200B amplifier (Molecular Devices) and digitized by Digidata 1322A (Molecular Devices). Synaptic stimulation was induced using the digital stimulator PG4000A (Cygnus Technology) and the stimulus isolator A365 (World Precision Instruments). Recordings were rejected when series resistance or holding current changed by 10%. Electrophysiology data are presented as mean±s.e.m. Given the possibility of variation, sEPSCs were recorded in multiple slices from a minimum of five mice in each group and pooled together for analysis using *t*-test. Repeated measure ANOVA followed by Bonferroni-corrected *post hoc* tests were used for comparing EPSCs. Greenhouse–Geisser correction was applied when sphericity was violated. *P*<0.05 was considered statistically significant.

### Behaviour

*Animals*. Male, C57BL6/J mice were used for all experiments. To eliminate potential developmentally sensitive, shipping-induced stress effects, breeding pairs of C57BL6/J wild-type mice from Charles River (Wilmington, MA) were set-up in the colony and monitored daily. Litters were weaned at P21 and males from various litters were randomly combined to eliminate any litter-driven effects on behaviour. Mice were housed five per cage in a temperature and humidity-controlled vivarium maintained on a 12 h light/dark cycle. Mice had *ad libitum* access to food and water. Separate cohorts of mice (aged P29–P90) were used for all fear conditioning, immunohistochemistry and electrophysiology experiments. All procedures regarding animal care and treatment were in compliance with guidelines established by Weill Cornell Medical College's Institutional Animal Care and Use Committee and the National Institutes of Health.

*Fear conditioning*. Mice were fear conditioned in mouse test cage (Coulbourn Instruments, Allentown, PA) inside a sound-attenuated box. The chamber (Context A) was cleaned in between each mouse and scented by peppermint-scented (0.1%) ethanol (EtOH; 70%). On conditioning day, (day 1), following a 2-min acclimation period, mice were conditioned with three trials consisting of a 30-s tone (5 kHz, 70-dB) that co-terminated with a 1-s, 0.7-mA foot shock delivered through the electrified floor grid. Each trial was separated by a 30-s inter-trial interval. After the final tone-shock pairing, mice remained in the conditioning chamber for 1 min before being returned to their home cages. Trials were digitally recorded and freezing during the initial 2 min acclimation/exploration period was scored as a measure of baseline freezing to the conditioning context.

For contextual reconsolidation update experiments, mice were returned to Context A for either a long-term memory test (LTM Test #1), or a retrieval session, 24 h post-conditioning, where freezing behaviour was scored during the last 3.5 min of the total 5.5 min spent in the chamber for contextual experiments. After this fear retrieval, mice were returned to their home cages and then returned to Context A for a contextual extinction session after a specified amount of time (10 min, 1 h, 24 h and so on. based on experimental group) and remained in the context for 1 h. During the post-extinction long-term memory (LTM Test #2) test, mice were returned to Context A again and freezing behaviour was scored during the last 3.5 min of a 5.5-min trial. To test for fear erasure by examining reinstatement, mice were introduced to a novel context (Context C; red cylinder, cleaned and scented with lemon scented (0.1%) EtOH (70%), with electrified floor grid), and after a 1-min acclimation period, were given a single, unsignaled (no tone cue) 1 s, 0.7-mA foot shock and remained in Context C for 1 min post-shock before being returned to their home cages. A final LTM test (LTM Test #3) was given in which mice were tested for their freezing to either tone (Context B; three 30 s tone cues) or context (Context A; last 3.5 min of a 5.5-min trial). Experiments that tested for erasure by examining fear renewal (day 4), examined freezing to Context A after a 1-month interval and measure freezing behaviour for the last 3.5 min of a 5.5-min trial.

Cue reconsolidation update experiments followed a similar format, except involved tone cue presentations. Following fear conditioning, mice were returned to their home cages. 24 h post-conditioning, mice were placed in Context B for a retrieval session during which a single tone cue was played. After this fear retrieval, mice were returned to their home cages and then returned to Context B for a cue extinction session after a specified amount of time (10 min, 1 h, 24 h and so on. based on experimental group) and were presented with 18 tones (unless noted otherwise in figures), with a 3-min intertrial interval. During the post-extinction long-term memory (LTM Test #2) test, mice were returned to Context A again and freezing to tone cues was scored and calculated as a percentage of time spent freezing to tone. To test for fear erasure by examining renewal, mice were remained in their home cages for over a month and re-tested for cue fear. To test for fear erasure by reinstatement, mice were introduced to a novel context (Context C; red cylinder, cleaned and scented with lemon scented (0.1%) EtOH (70%), with electrified floor grid), and after a 1-min acclimation period, were given a single, unsignalled (no tone cue) 1 s, 0.7-mA foot shock and remained in Context C for 1 min post-shock before being returned to their home cages and then subsequently tested for erasure by a post-reinstatement cue test.

For context-cue interaction studies and to see if the context in which tone cue extinction is presented has an effect on subsequent behaviour, mice were fear conditioned (Context A) and then split into four groups: one group remained in their home cage and did not experience any extinction tones; one group received contextual extinction only (as described above); one group underwent tone cue extinction in a novel context (Context B); one group underwent tone cue extinction in the fear-conditioning context (Context A). Mice were then re-tested for their cue fear in the context as specified in each figure, after a 2-week period. 24 h post-LTM, attempts were made at re-extinguishing this fear to tone (Context B), with 10 tone presentations with a 3-min intertrial interval. To test for effects of age, we used a one-way ANOVA. To assess effects of test phase, we used a general linear model repeated measures ANOVA, with *post hoc* corrections for pairwise comparisons. For behavioural studies, to eliminate potential effects of litter size or quality of early care on behavioural outcomes, each developmental time point included animals from a minimum of two separate litters, and in some cases up to four different litters.

## Additional information

**How to cite this article:** Pattwell, S. S. *et al*. Dynamic changes in neural circuitry during adolescence are associated with persistent attenuation of fear memories. *Nat. Commun.* 7:11475 doi: 10.1038/ncomms11475 (2016).

## Supplementary Material

Supplementary Information Supplementary Figures 1-14, Supplementary Tables 1-2, Supplementary Notes 1-2 and Supplementary References
