# Supplementary material for: Dynamic changes in neural circuitry during adolescence are associated with persistent attenuation of fear memories
Source: Nat Commun. 2016 May 24;7:11475. doi: 10.1038/ncomms11475 (PMC4890178; doi:10.1038/ncomms11475)
Supplement: Supplementary Information — Supplementary Figures 1-14, Supplementary Tables 1-2, Supplementary Notes 1-2 and Supplementary References [file ncomms11475-s1.pdf]

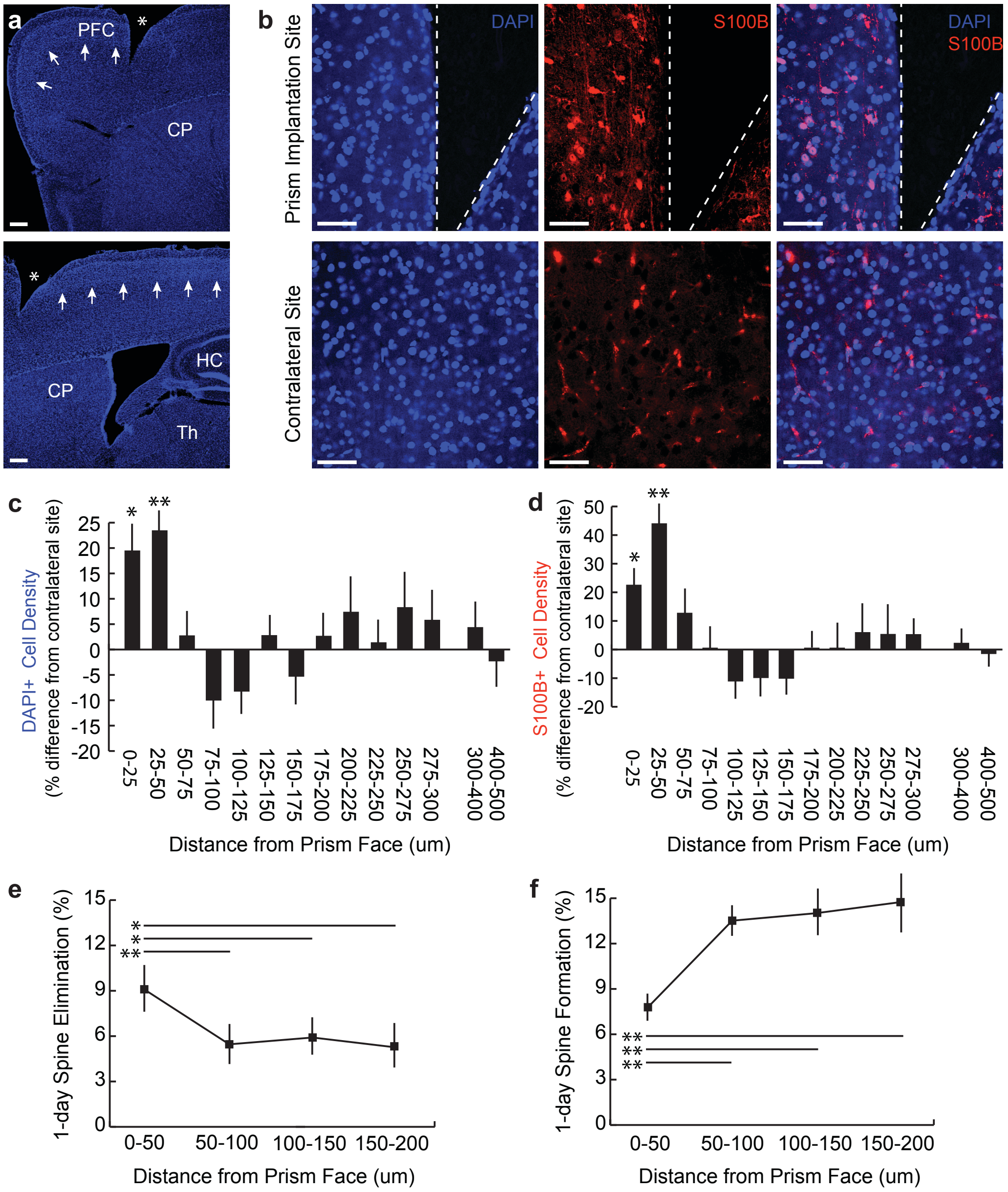

Supplementary Figure 1. Preservation of cortical lamination and neuroanatomical structures adjacent to prism implant. (a) Cortical lamination patterns (arrows) and neuroanatomical structures adjacent to the prism implant (denoted by asterisk) were grossly intact in these low-magnification images of sagittal sections stained for DAPI. PFC = prefrontal cortex. CP = caudate-putamen. HC = hippocampus. Th = thalamus. Scale bar = 300 μm. **(b)** High-magnification images (40X objective) of the prism

implantation site stained for DAPI and S100B a marker of mature glial cells, approximately one week after prism implantation. Images from the corresponding area of PFC in the contralateral hemisphere are included for comparison. Dashed lines denote contours of the prism. Scale bar = 50 µm. **(c-d)** Cell density (DAPI+; panel **C;** Kruskal Wallis K = 32.8, P = 0.003) and gliosis (S100B+; panel **d**; Kruskal Wallis K = 33.8, P = 0.002) varied significantly with distance from the prism face, plotted here relative to control measurements (% difference) obtained from the contralateral site. 5–7 days after prism implantation at ~P24 (i.e. after a delay comparable to the recovery period used for all *in vivo* imaging experiments), there was a modest but significant increase in cell density and gliosis at distances of 0–25 µm and 25–50 µm from the prism face, relative to the contralateral site. There were no significant changes in cell density or gliosis at distances of 50–500 µm from the prism face. * = P < 0.05, ** = P < 0.01, by Kruskal-Wallis ANOVA with post-hoc contrasts (Dunn's Test), relative to densities in the contralateral hemisphere. **(e)** 1-day spine elimination (Kruskal Wallis K = 9.01, p = 0.029) and **(f)** 1-day spine formation rates (K = 15.8, P = 0.001) in dorsal mPFC / prelimbic cortex (P30–P31), plotted as a function of distance from the prism face. Spine elimination was modestly but significantly increased, and spine formation was decreased, at distances of 0–50 µm from the prism face, compared to 150–200 µm from the prism face. * = P < 0.05, ** = P < 0.01, for post-hoc contrasts (Dunn's Test). All error bars = SEM.

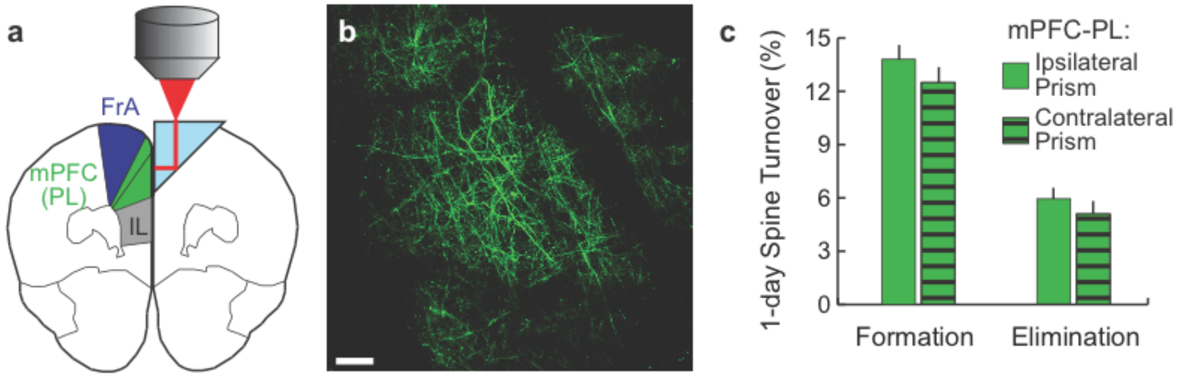

Supplementary Figure 2. Preparation for mPFC imaging. (a) Schematic of preparation for imaging mPFC across the midline, through microprisms implanted in the contralateral hemisphere, using a method adapted from Low et al.,[1]. This preparation allowed us to image spine turnover without affecting projections to and from mPFC in the ipsilateral hemisphere. As in the main text, the majority of dendritic segments that were analyzed were for cells that reside in prelimbic cortex (PL), although some may reside in the neuroanatomically related cingulate area of mPFC, which lies dorsal to PL. Infralimbic (IL) dendrites were excluded because they lie ventral to the face of the prism. FrA = frontal association cortex (excluded from this analysis). **(b)** Representative image depicting the distal aspects of apical dendrites projecting to Layer 1 from yellow fluorescent protein (YFP)-expressing pyramidal cells in the PL area of mPFC. The dark bands are blood vessels, which obscure the underlying dendrites. Scale bar = 50 µm. **(c)** 1-day spine formation and elimination rates (P30–P31) were not significantly different ($P > 0.38$, Mann-Whitney) when quantified through a prism implanted in the contralateral hemisphere, compared to those quantified through an ipsilaterally implanted prism and reported in the main text. Error bars = SEM.

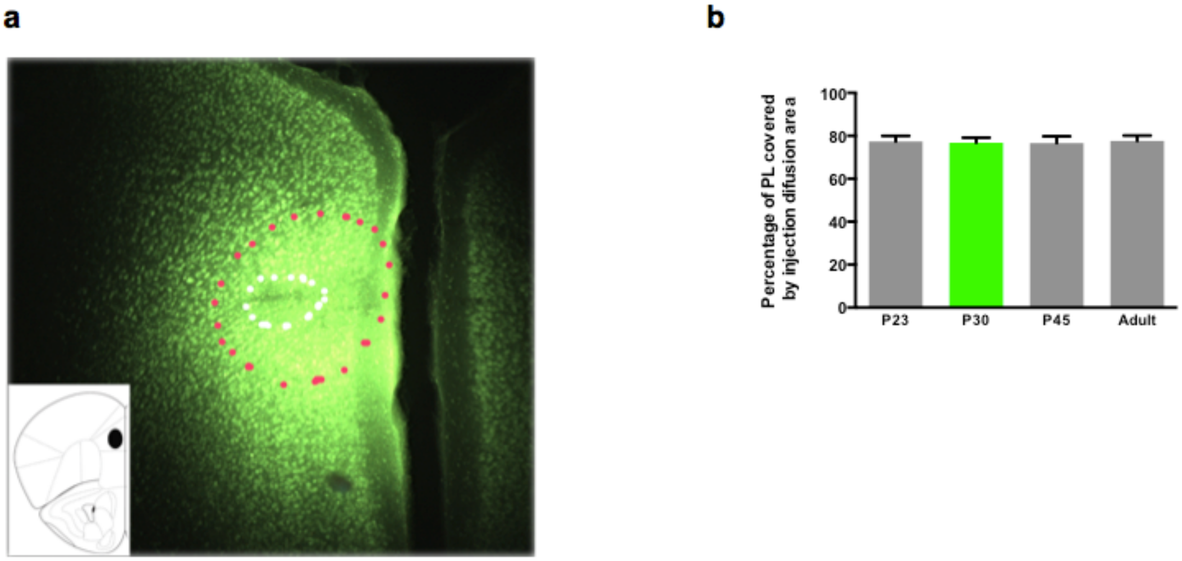

Supplementary Figure 3. Fluorogold injection site and diffusion through PL. **(a)** Typical injection site located in the rostral portion of PL, including an injection center (white dash line circle) and diffusion area (red dash line circle) and adjacent retro-labeling area. Lower left inset showing the injection site in the anatomical map**. (b)** In order to rule out the effects of topographic projection difference, only the cases in which injection centers located in rostral portion of PL were included. The area of PL and area covered by the injection (injection center + diffusion area) were estimated separately using Cavalieri's estimation. We then calculated the percentage of PL covered by the injection area by dividing the area covered by the injection by the total PL area. No differences were observed between the four groups based on absolute volume, one-way ANOVA ($F_{(3,16)}$ = 0.03190, p = 0.9920). Brown-Forsythe and Bartlett's tests also show that there is no significant difference between groups.

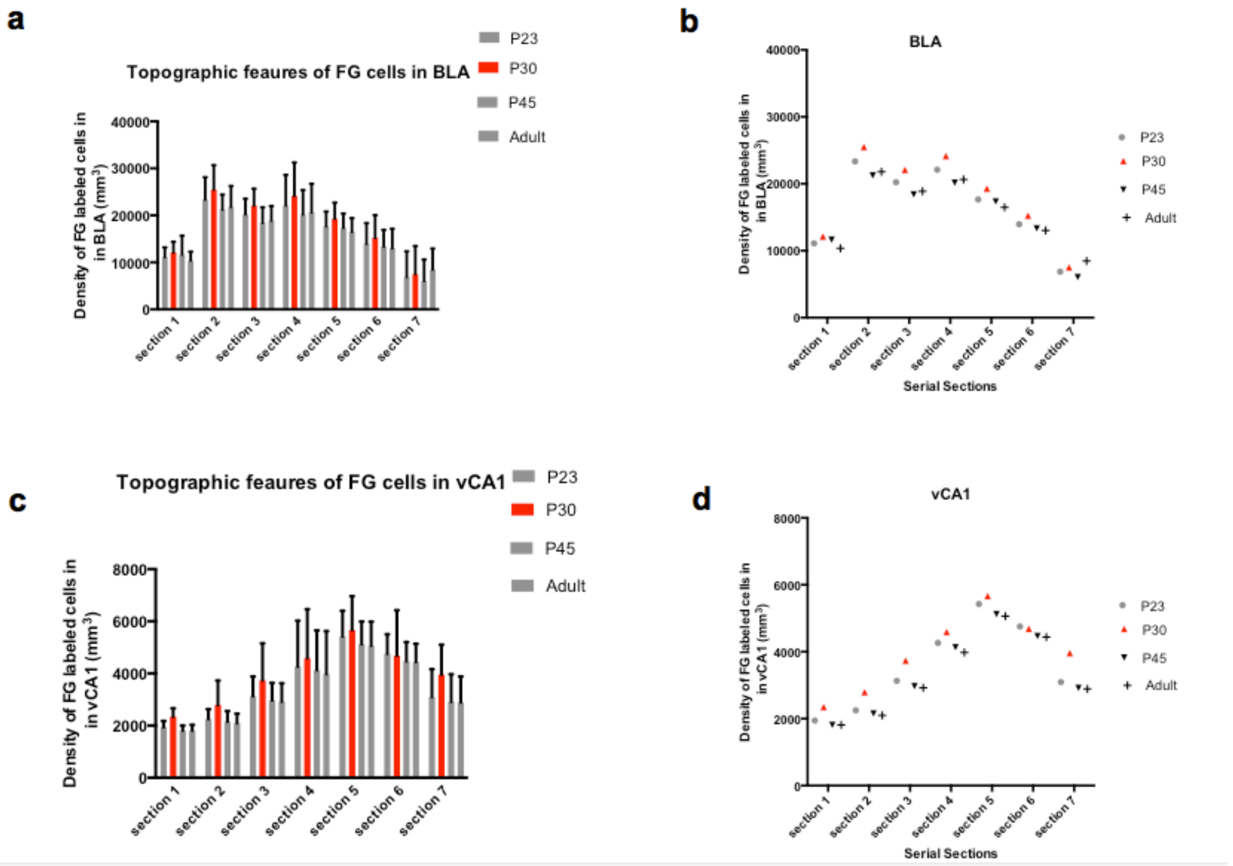

Supplementary Figure 4. Topographic features across development. Although injection sites cover most of the PL, possible topographic projections may affect numbers of labeled neurons in BLA and vCA1. We then analyzed the topographic features and compared them across different ages. Patterns within BLA (**a, b)** and vCA1 **(c, d)** are consistent across different ages displaying similar topographic features - densest labeled neurons were observed in middle 1/3 of BLA, while vCA1 has the most densely labeled neurons in its caudal 1/3.

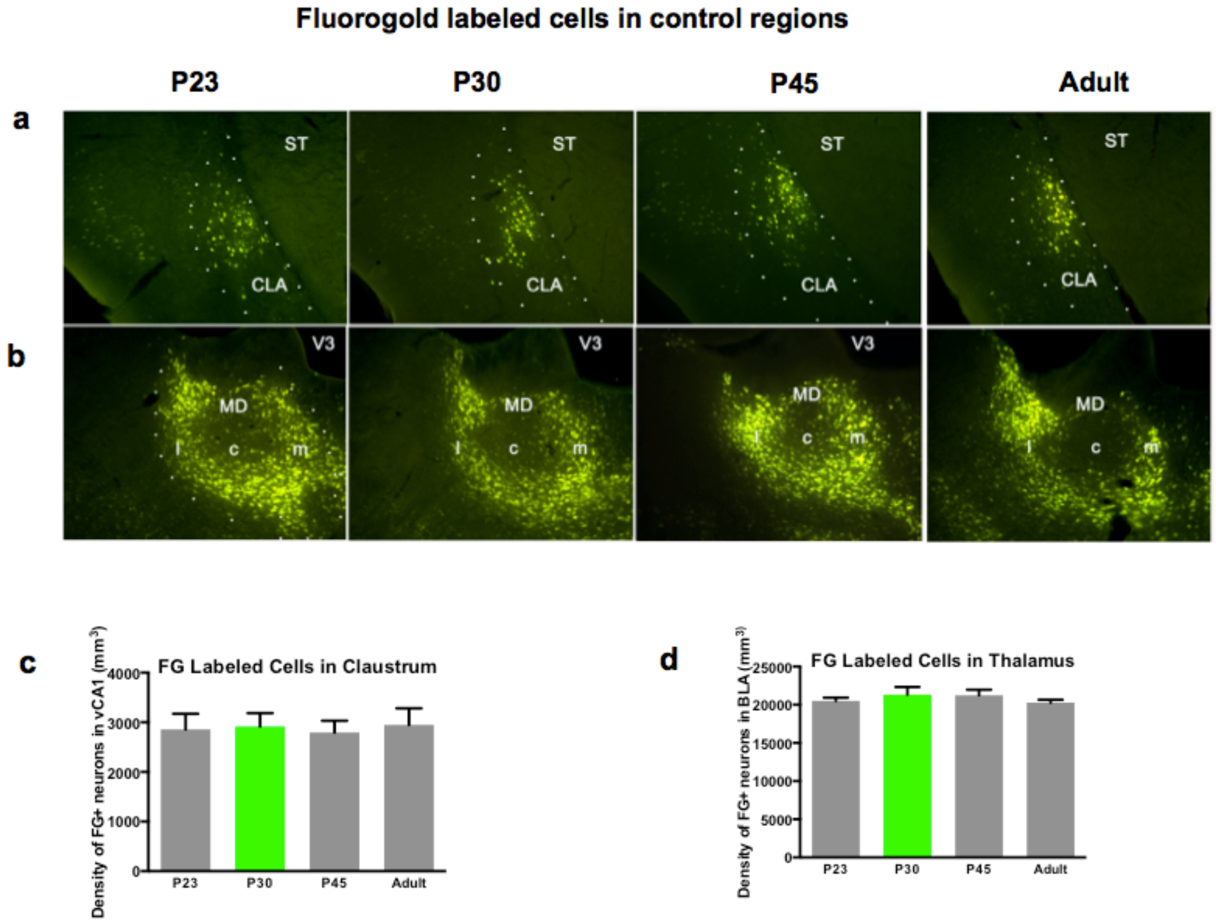

Supplementary Figure 5. Fluorogold labeling in control regions across development. Fluorogold labeling in control regions **(a)** claustrum and **(b)** thalamic nuclei, shows consistency in other brain regions after PL injection. There are no developmental differences in **(c)** claustrum (F (3,12) = 0.05291, P = 0.9832) or **(d)** thalamic nuclei (F (3,12) = 0.5862, P = 0.6355).

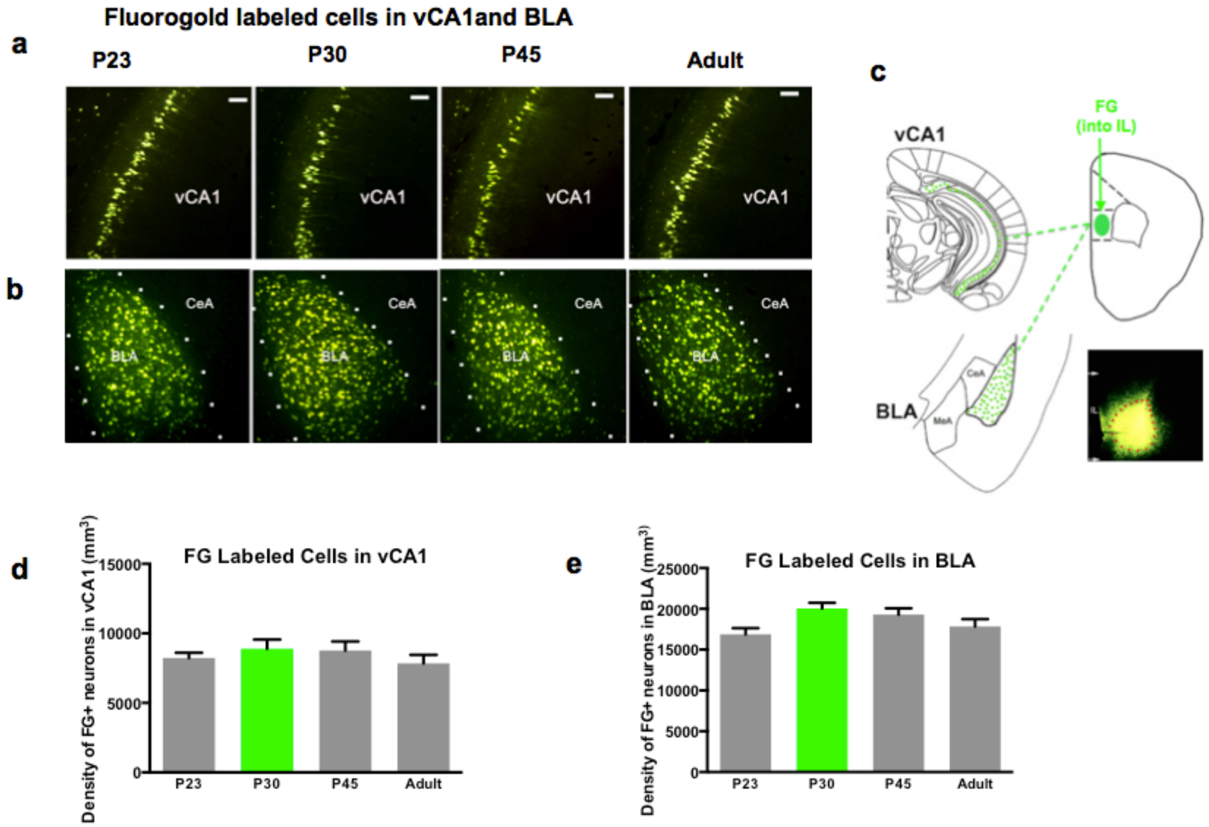

Supplementary Figure 6. Fluorogold labeling in vCA1 and BLA from IL injections. Representative images of fluorogold labeled cells in **(a)** hippocampus vCA1 and **(b)** basolateral amygdala (BLA), 10X resulting from injections in infralimbic cortex. **(c)** Schematic of fluorogold (FG) injections in infralimbic cortex (IL) and FG+ cells throughout vCA1 and BLA. Outlined area denotes injection site, with surrounding diffusion maintained within IL boundaries. Using quantification of the density of fluorogold (FG+) labeled neurons, we found no differences between groups in the density of labeling in **(d)** vCA1, one-way ANOVA ($F_{(3,12)}$ = 0.6919, *P* = 0.5744), (P23 8232.67 ± 370.26; P30 8891.33 ± 661.67; P45 8771.55 ± 645.84; P90 7847.98 ± 608.64) or **(e)** BLA, one-way ANOVA, ($F_{(3,12)}$ = 3.350, *P* = 0.0556) P23 16875.63 ± 751.94; P30 20042.61 ± 698.86; P45 19303.34 ± 772.26; P90 17851.96 ± 877.95).

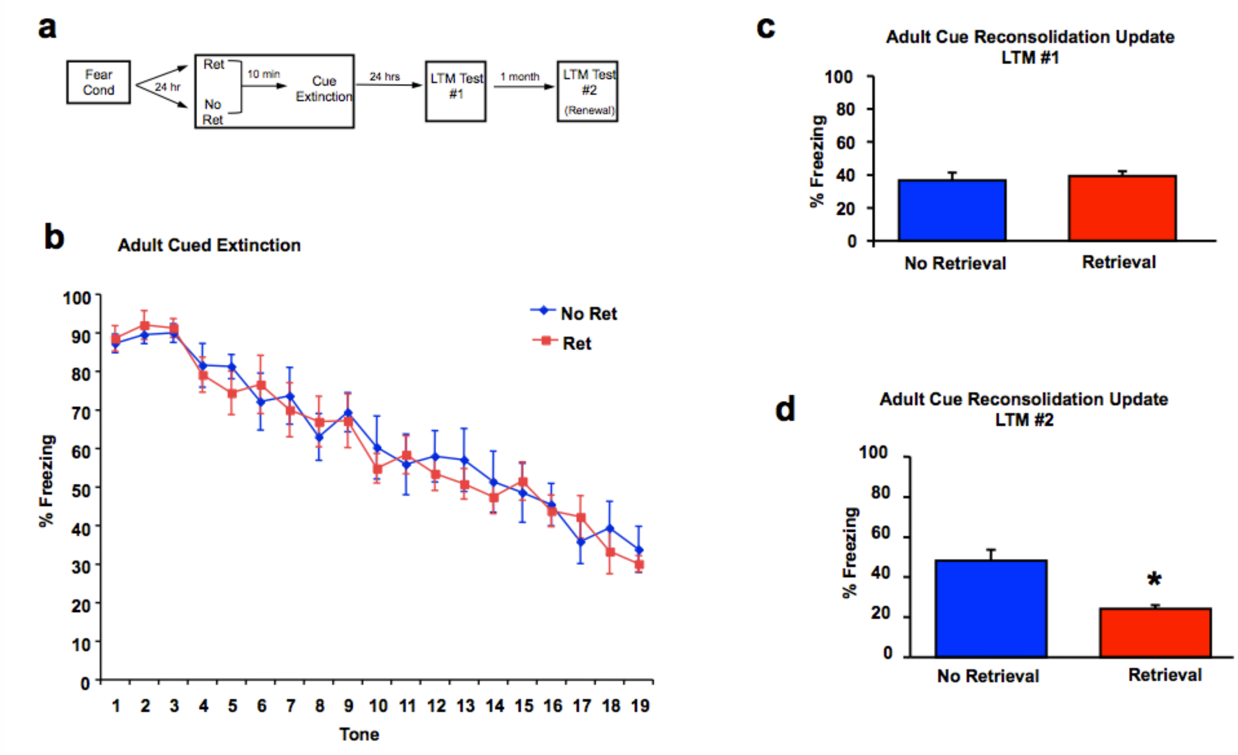

Supplementary Figure 7. Adult cued fear erasure. (a) Behavioral paradigm for adult cued fear memory erasure outlines fear conditioning on day 1, followed by a cue retrieval 10 minutes prior to cued extinction on day 2, with subsequent long-term memory (LTM) cue tests 24 hrs and 1 month later. **(b)** There was no significant difference during extinction learning between Retrieval (Ret) or No Ret groups, or **(c)** during the post-extinction LTM Test #1 [No Ret 40.33% ± 3.83; Ret 37.65% ± 3.02]. **(d)** Ret mice showed significantly decreased freezing behavior during the renewal erasure test, LTM Test #2, indicative of fear erasure as a result of the persistent fear attenuation paradigm [No Ret 48.01% ± 5.71; Ret 24.17% ± 1.76, $P < 0.05$]. Ret, Retrieval. All results are presented as a mean ± SEM determined from analysis 8 – 12 mice per group.

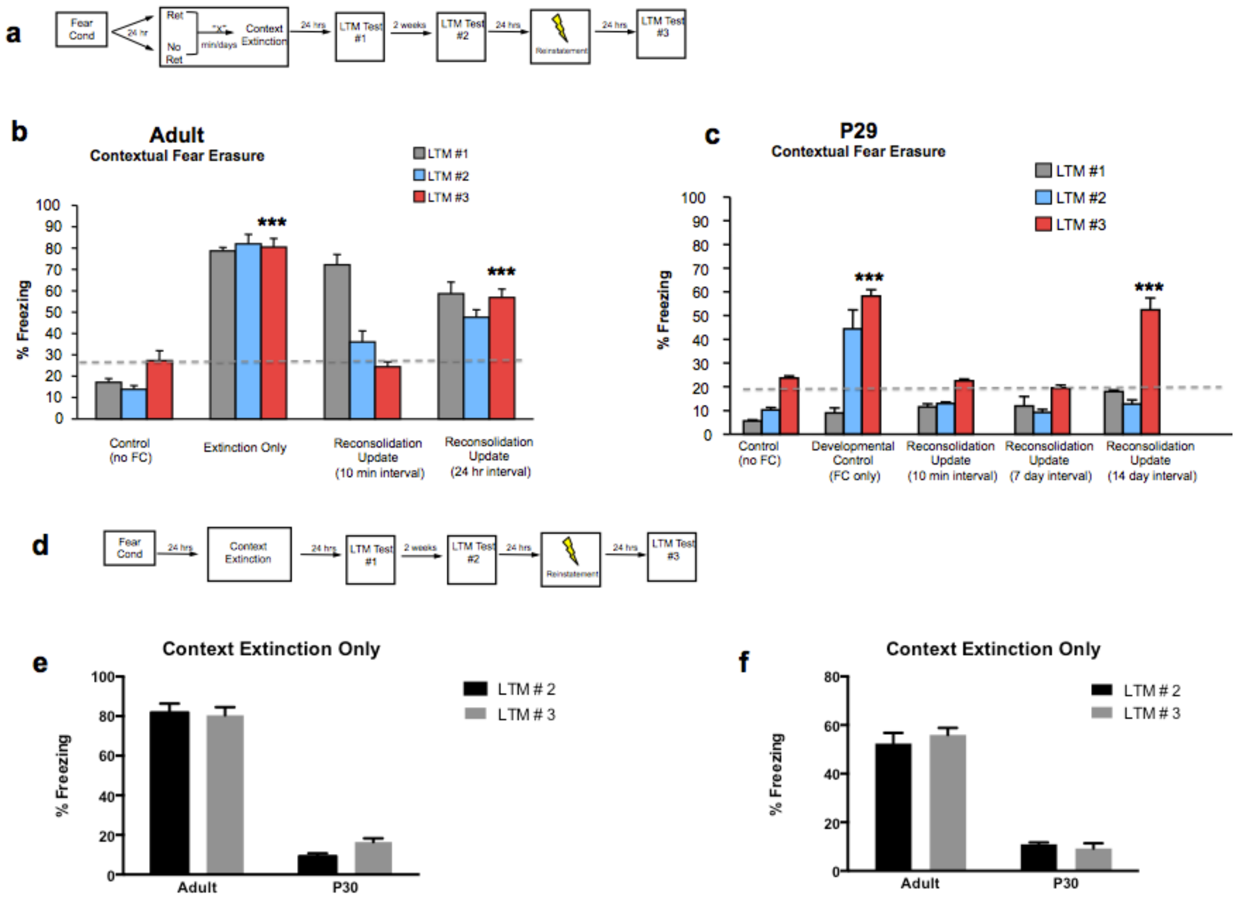

Supplementary Figure 8. Contextual fear erasure in adult and adolescent mice. (a) Behavioral paradigm for adult and adolescent contextual erasure outlines fear conditioning on day 1 followed by a contextual retrieval session at various intervals (data shown in B) prior to contextual extinction on day 2, with subsequent long-term memory (LTM) context tests both 24 hrs and 14 days later, along with reinstatement and a final LTM context test 24 hr post-reinstatement. **(b)** Adult mice that underwent contextual extinction 10 min after contextual retrieval showed equivalent levels of post-reinstatement freezing to non-fear conditioned controls, 2 weeks later, indicative of contextual fear erasure. **(c)** P29 mice that underwent contextual extinction within (10 min) or outside (7 days) showed equivalent levels of post-reinstatement freezing to non-fear conditioned controls, indicative of contextual fear erasure, as long as contextual extinction occurred prior to P42. All results are presented as a mean ± SEM determined from analysis 8 – 12 mice per group. **(d)** Behavioral paradigm for contextual extinction. Mice were fear conditioned in Context A. 24 hours later all mice were given a one hour extinction session in Context A, followed by three 5.5 minute Long Term Memory (LTM) Tests at varying time points, including one post-reinstatement. Only LTM #2 and LTM #3 are displayed to avoid confounds associated with P29 sensitive period of contextual fear expression. **(e)** P29 fear conditioned mice who underwent contextual extinction 24 hr post-conditioning showed persistently attenuated contextual fear at P43 at LTM #2 and post-reinstatement at LTM #3, whereas adult contextual fear remained high

throughout both LTM #2 and LTM #3. *** = $P << 0.005$. **(f)** P29 fear conditioned mice that underwent contextual extinction 24 hr post-conditioning showed persistently attenuated contextual fear at P43 at LTM #2 and post-reinstatement at LTM #3, when freezing behavior was measured for the entire time in context, whereas adult contextual fear remained high throughout both LTM #2 and LTM #3. *** = $P << 0.005$

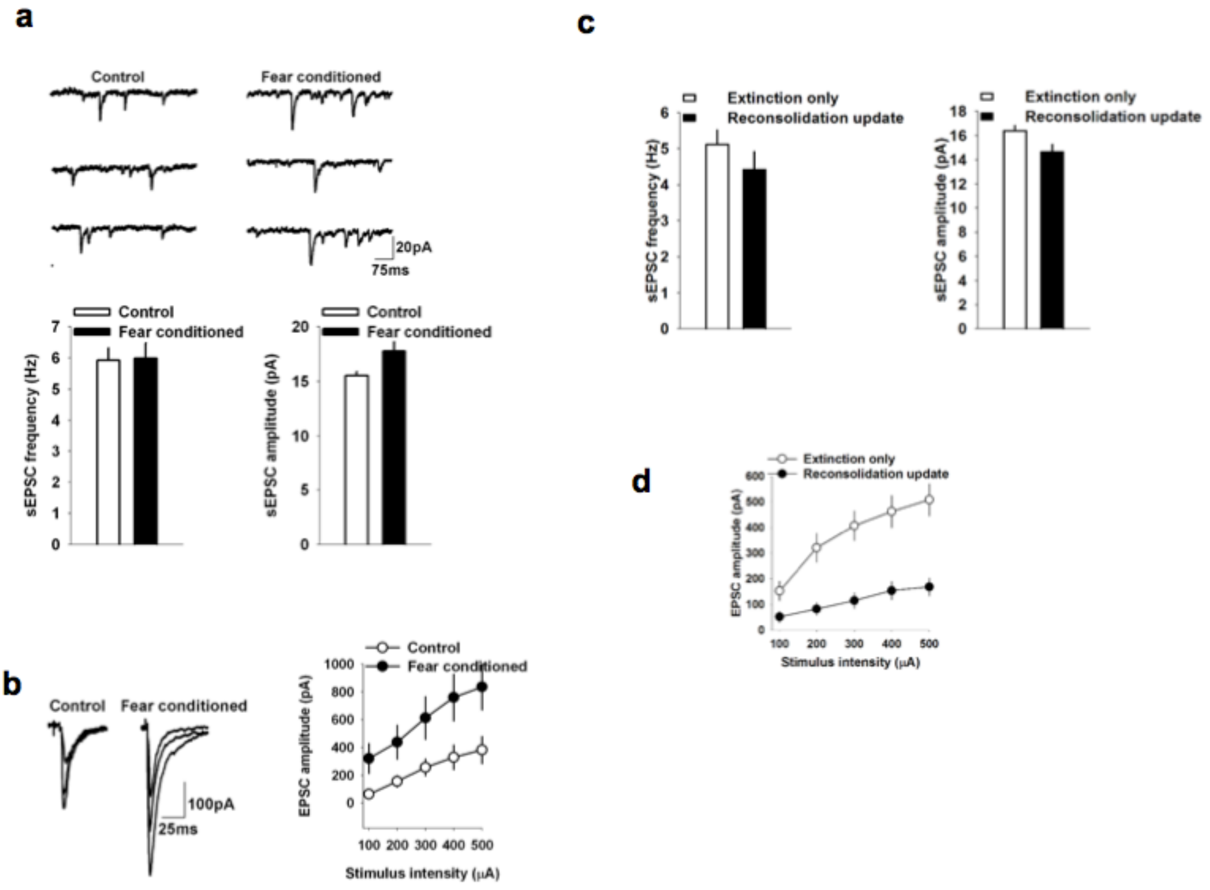

Supplementary Figure 9. Contextual fear conditioning involves potentiation of excitatory synaptic transmission in the basal amygdala of adult mice. (a) Average sEPSC frequency and amplitude in the basal amygdala principal neurons of control and fear conditioned mice. The sEPSC amplitude ($t(48) = 2.22$, $P = 0.03$) but not the frequency ($t(48) = -0.1$, $P = 0.92$) was significantly higher in fear-conditioned mice. Upper panel shows examples of sEPSC recordings from control and fear conditioned mice. **(b)** Average EPSC amplitude in the basal amygdala principal neurons of control and fear conditioned mice. A significant effect of group ($F(1,37) = 5.4$, $P = 0.026$), stimulus intensity ($F(1.26,46.5) = 31.4$, $P < 0.001$) and a non-significant interaction between factors ($F(1.26,46.5) = 2$, $P = 0.16$) were observed. Left panel shows examples of EPSC recordings from control and fear conditioned mice. **(c)** Average sEPSC frequency and amplitude in the basal amygdala principal neurons from mice that had undergone fear extinction alone and fear extinction following a prior reminder cued. The sEPSC amplitude ($t(40) = 2.48$, $P = 0.018$) but not the frequency ($t(40) = 1.1$, $P = 0.27$) was significantly decreased in mice that had undergone fear extinction following a prior reminder cued. **(d)** Average EPSC amplitude in the basal amygdala principal neurons from mice that had undergone fear extinction alone and fear extinction following a prior reminder cued. A significant effect of group ($F(1,41) = 18.5$, $P < 0.001$), stimulus intensity ($F(1.53,62.9) = 33.5$, $P < 0.001$) and a significant interaction between factors ($F(1.26,46.5) = 8.46$, $P = 0.001$) were observed.

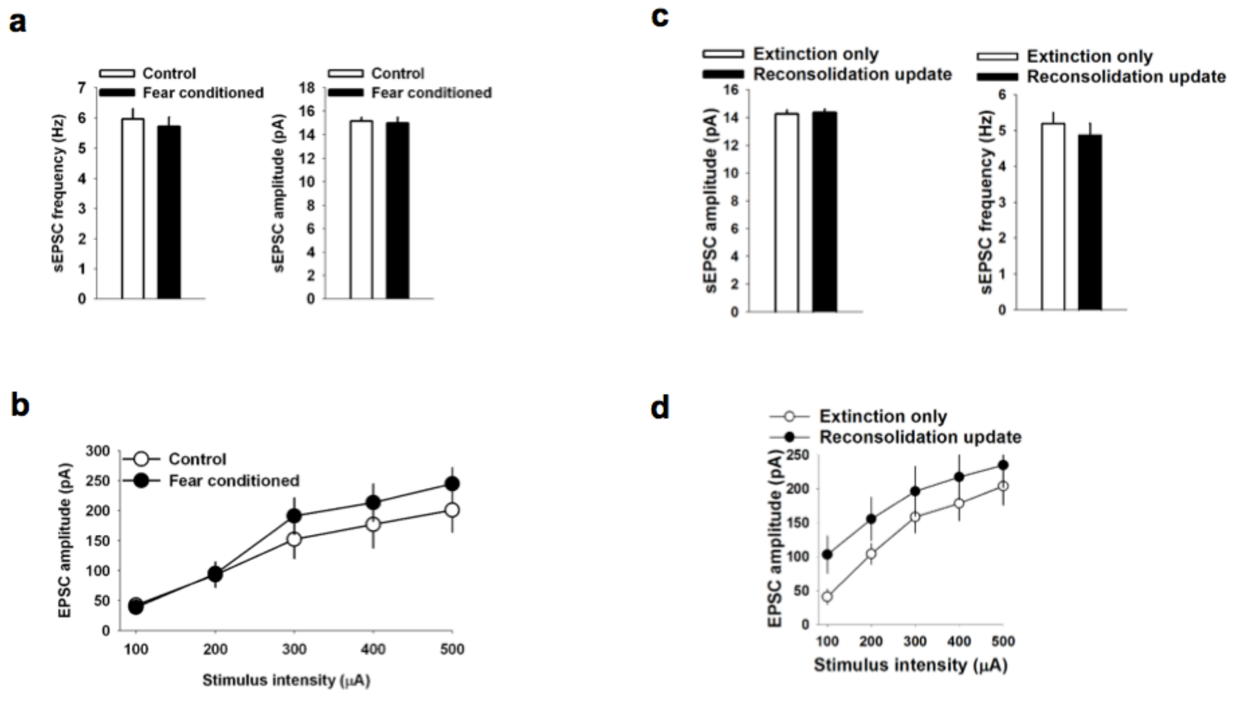

Supplementary Figure 10. **Fear conditioning did not affect excitatory synaptic transmission in the basal amygdala in adolescent mice. (a)** Average sEPSC frequency and amplitude in the basal amygdala principal neurons of control and fear conditioned P29 mice. The sEPSC frequency (t(37) = -0.54, *P* = 0.58) and amplitude (t(37) = -0.24, *P* = 0.81) were not significantly different between control and fear conditioned mice. **(b)** Average EPSC amplitude in the basal amygdala principal neurons of control and fear conditioned P29 mice. A non-significant effect of group (F(1,29) = 0.54, *P* = 0.47), significant effect of stimulus intensity (F(1.59,46.03) = 43.6, *P* < 0.001) and a non-significant interaction between factors (F(1.59,46.03) = 0.97, *P* = 0.37) were observed. **(c)** Average sEPSC frequency and amplitude in the basal amygdala principal neurons from P29 mice that had undergone fear extinction alone and fear extinction following a prior reminder cued. The sEPSC frequency (t(32) = 0.72, *P* = 0.48) and amplitude (t(32) = -0.25, *P* = 0.8) were not significantly different between mice that had undergone fear extinction alone and fear extinction following a prior reminder cued. **(d)** Average EPSC amplitude in the basal amygdala principal neurons from P29 mice that had undergone fear extinction alone and fear extinction following a prior reminder cued. A non-significant effect of group (F(1,34) = 1.5, *P* = 0.23), significant effect of stimulus intensity (F(1.58,53.8) = 50.3, *P* < 0.001) and a non-significant interaction between factors (F(1.58,53.8) = 0.6, *P* = 0.54) were observed.

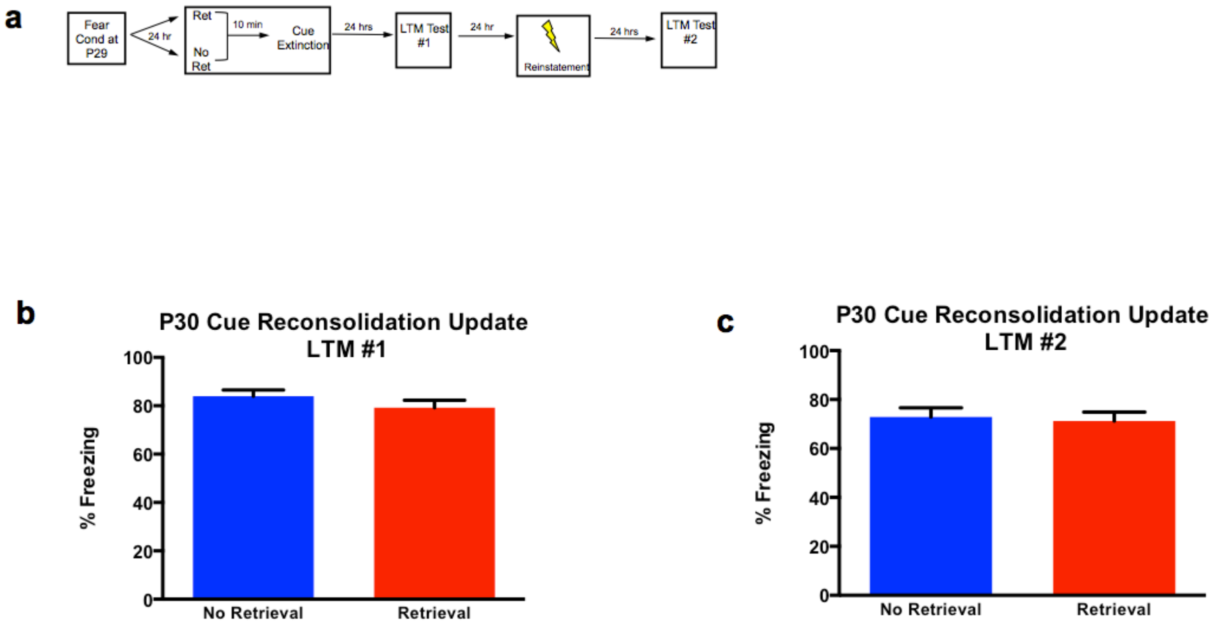

Supplementary Figure 11. **Adolescent cued fear erasure. (a)** Behavioral paradigm for P29 cued fear memory erasure, modified from adult paradigm to account for sensitive period of contextual fear suppression. **(b)** There was no significant difference during extinction learning between Ret or No Ret groups, as shown by averages of the first three and last three extinction tones during LTM #1. **(c)** There was no significant difference during LTM #2 between Ret or No Ret groups, $P > 0.05$.

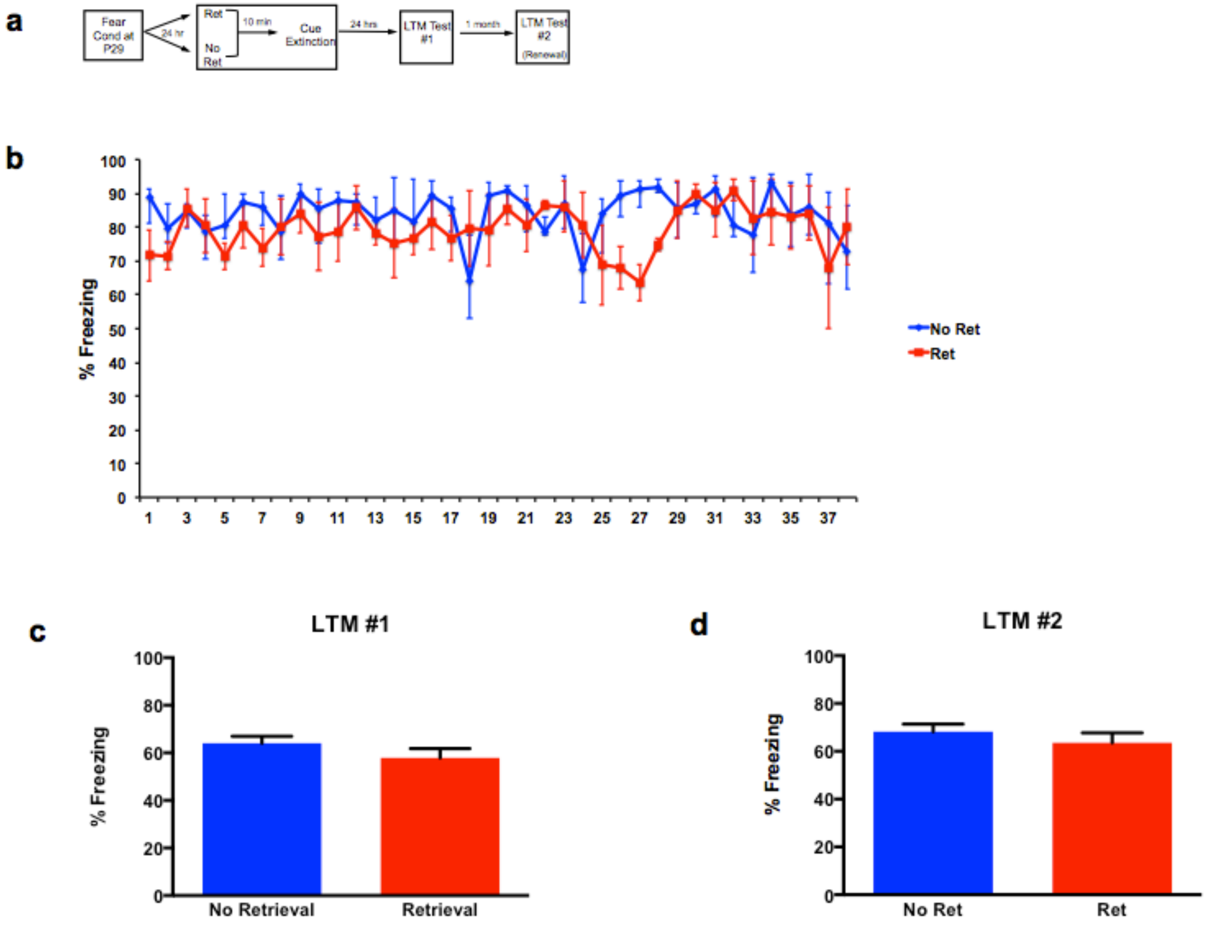

Supplementary Figure 12. Extended extinction paradigm for adolescent mice. Behavioral paradigm **(a)** and corresponding behavioral data **(b)** for P29 cued fear erasure via a reminder cue and extinction session of 38 tones with 3 min ITIs. There was no significant difference during extinction learning between Ret or No Ret groups **(b)**, at LTM #1 **(c)**, or at LTM #2 **(d)**, $P > 0.05$.

a

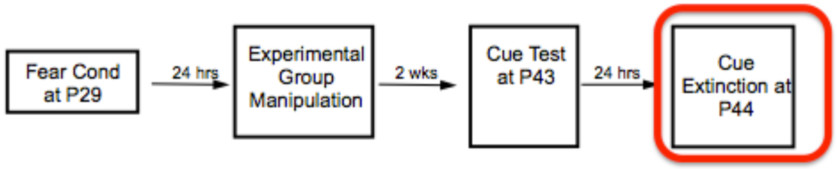

b

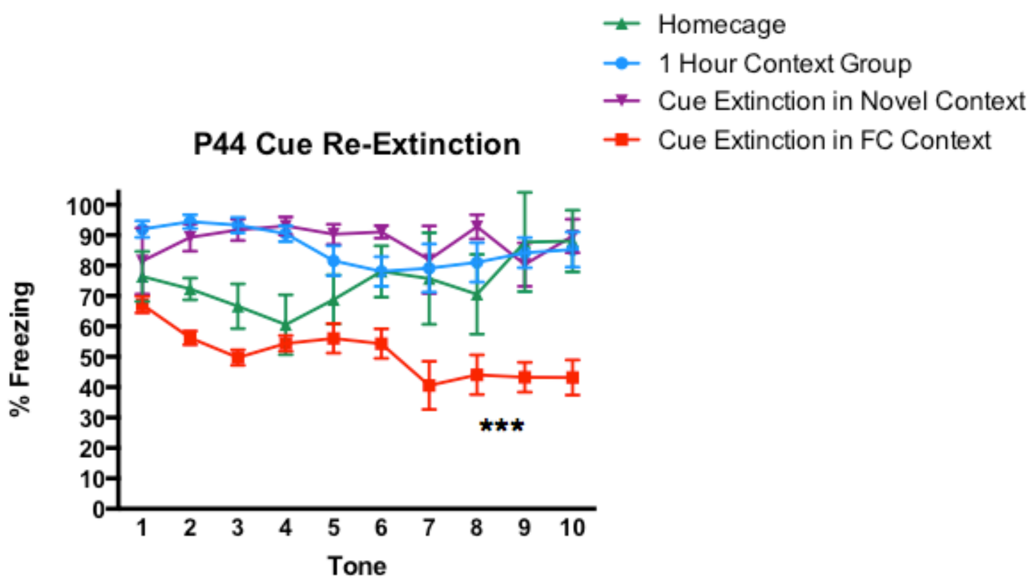

Supplementary Figure 13. Combined contextual and cued extinction effects on long term memory. (a) Behavioral paradigm depicting the timeline of Re-Extinction session for groups outlined in Main Figure 3. **(b)** Re-extinction for all four behavioral treatment groups demonstrates significantly less freezing in the group that underwent cue fear extinction in the fear conditioning context ($F(3,12) = 24.1$, $P < 0.0001$). N=4 per group. All results are presented as a mean ± SEM determined from analysis 4 mice per group. *** = $P < 0.005$.

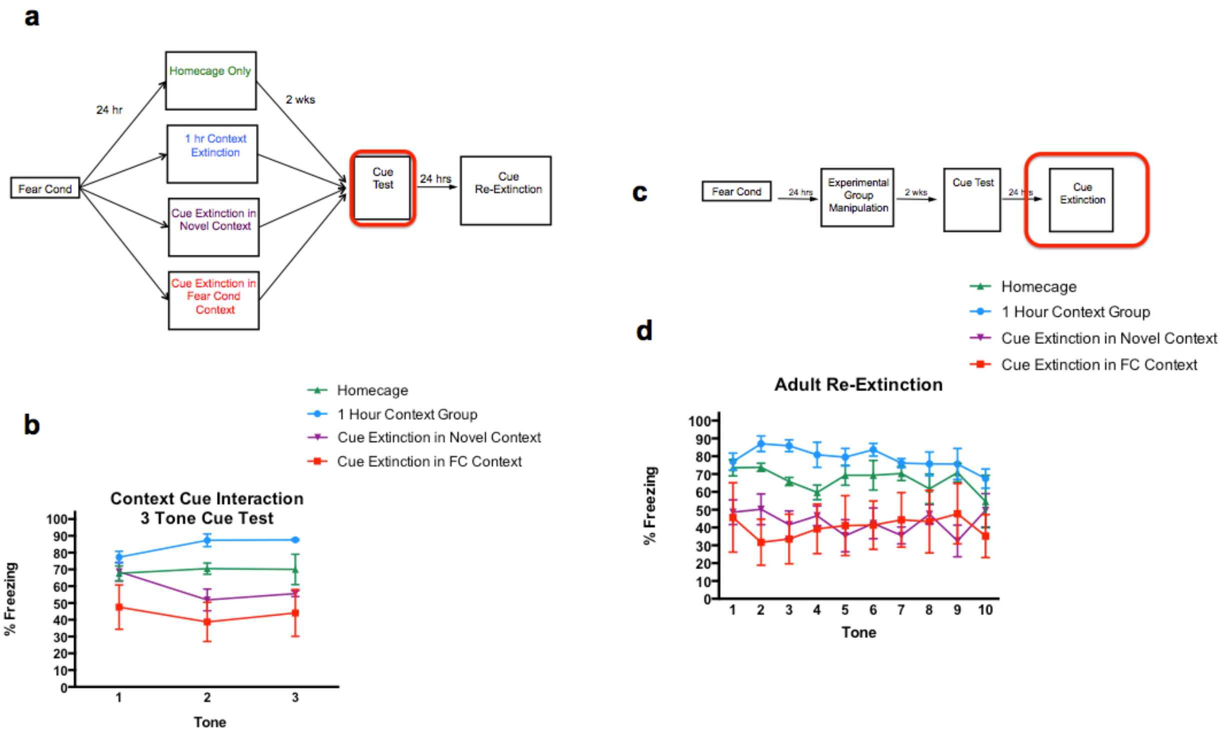

Supplementary Figure 14. Combined contextual and cue extinction effects in adult mice. (a) Behavioral paradigm depicting four groups designed to assess if there is an interaction between cued fear extinction and the context in which it is presented for adult fear conditioned and then tested at a later time point. **(b)** Three tone test for all four behavioral treatment groups demonstrates significantly less freezing in the group that underwent cued fear extinction in the fear conditioning context ($F(3,12) = 5.679$, $P = 0.0117$). **(c)** Behavioral paradigm depicting the timeline of Re-Extinction session for groups outlined in **(a)**. **(d)** Re-Extinction for all four behavioral treatment groups demonstrates significantly less freezing in the group that underwent cued fear extinction in the fear conditioning context ($F(3,12) = 5.356$, $P = 0.0142$). N=4 per group.

Supplementary Table 1.
Coordinates for FG mPFC injection (mm) – Prelimbic cortex

	AP	ML	DV
P23	1.90	0.40	1.80
P30	1.95	0.50	1.95
P45	1.98	0.50	1.98
Adult	1.98	0.50	1.98

Supplementary Table 2.
Coordinates for FG mPFC injection (mm) – Infralimbic cortex

	AP	ML	DV
P23	1.60	0.35	2.30
P30	1.65	0.35	2.40
P45	1.70	0.40	2.50
Adult	1.70	0.40	2.50

Supplementary Notes

Supplementary Note 1. Developing a contextually based behavioral protocol for persistent attenuation of fear memories

Modification of existing cued-fear paradigms led to development of a protocol that relies on contextual retrieval and contextual extinction to attenuate fear memories in adolescent and adult mice. Through modification of existing cued-erasure paradigms that draw upon principles of persistent attenuation of memories, we implemented a contextual-based behavioral protocol tailored to persistently attenuate contextual fear memory in adult mice (Supplementary Figures 7-8)[2,3]. After confirming that there were no inherent baseline differences in locomotor acitvity or altered capacity for fear conditioning and freezing behavior across the various age groups, we sought to test whether these contextual paradigms that proved efficacious in adult mice may be effective during adolescence. We fear conditioned mice during early adolescence (P29) and exposed them to the same persistent contextual fear attenuation paradigm as the adults (shown in Supplementary Figure 8). Because contextual fear expression during these early adolescent ages is minimal, we were interested in how an early-life contextual extinction session may influence later behavior, when contextual fear expression is known to re-emerge[4]. Interestingly, mice that underwent persistent contextual fear attenuation training at P29 showed significant decreases in freezing behavior when tested two weeks later (Supplementary Figure 8), indicating that the delayed expression of contextual fear memory that emerges during adulthood[4] was prevented by the prior persistent contextual fear attenuation procedure. By intervening early in life, at a time when the mice do not behaviorally express contextual fear memory, we show that we can prevent the fear from ever being expressed. This finding suggests that a conditioned aversive memory can be persistently altered via behavioral intervention, similar to pharmacological studies with brain-derived neurotrophic factor (BDNF) in which fear memories are eliminated in the absence of any extinction sessions[5].

Because the suppression of contextual fear expression in adolescent mice lasts until circa P40 when sexual maturity is reached[4], we tested whether the window for intervention is broadened beyond the windows reported in persistent memory attenuation studies with adult rodents[2]. To test this hypothesis, separate cohorts of P29 mice were fear conditioned and given a single contextual retrieval session 24 hours later (Supplementary Figure 8). We then exposed separate cohorts of P30 mice to a one-hour extinction session at various intervals, and tested the mice for contextual fear expression during late adolescence at Long-Term Memory (LTM) Test # 2, and after reinstatement, at LTM # 3 (Supplementary Figure 8). Surprisingly, in contrast to the narrow time window during which memories can be attenuated in adults, we show a broadened window of intervention in adolescent mice that that coincides with the sensitive period during which contextual fear expression is minimal. This pattern was also true during post-reinstatement testing (Supplementary Figure 8). However, when the window between memory retrieval and subsequent extinction was broadened beyond P43, which coincides with developmental pruning of hippocampal-PL projections and the re-emergence of the capacity to express contextual fear memories[4],

the paradigm was ineffective in persistently attenuating fear memories (Supplementary Figure 8). These results demonstrate that there is a prolonged sensitive period during adolescence when contextual fear memories are particularly susceptible to persistent attenuation. During early adolescence when PL-hippocampal circuits are rewiring, contextual memories may be particularly fragile due to competing memory traces within the network. This interpretation is similar to recent work suggesting that neurogenic waves in the hippocampus can impair memories on certain tasks[6].

Supplementary Note 2. Susceptibility of contextual fear memory during sensitive period in adolescent mice

Contextual fear in adolescent mice is susceptible to persistent attenuation through contextual extinction, occurs regardless of a memory retrieval session and is supported by electrophysiological correlates in the basal amygdala. Principles of persistent fear attenuation rely heavily on the successful retrieval of the initial fear memory followed by a limited window of time during which fear memories are labile, yet, this window is significantly broadened during the adolescent sensitive period. Therefore, we tested if a retrieval session was needed to persistently attenuate the memory, or if a single, one-hour contextual extinction session would be sufficient to persistently attenuate contextual fear during adolescence. Previous work suggests that extinction in pre-weanling rodents leads to degradation of a weak memory trace and thus an "amnesia" for cued fear as evidenced by a lack of renewal or recovery in preweanling rodents, which has been shown to be dependent on GABAergic signaling and perineuronal net formation in post-weanling rodents[7-9]. It is plausible that a memory trace relying on a highly dynamic BLA-hippocampal-PL circuit may be susceptible to a similar mechanism of memory degradation during adolescence given the heightened plasticity of the circuit, specifically at this age. To test this hypothesis, we fear conditioned mice at P29, followed by a contextual extinction session the next day without any prior retrieval sessions (Supplementary Figure 8d,e). When these mice were tested during late adolescence, the absence of fear persisted. In contrast to the effects in adult mice, contextual fear was not reinstated after an unsignaled foot-shock, indicative of successful persistent attenuation of the fear memory or fear erasure (Supplementary Figure 8). These results suggest that adolescent contextual fear can be erased with or without employing retrieval cues associated with memory attenuation paradigms, as long as the intervention occurs during the sensitive period of P29-P43 (adolescence) in mice[4], These data highlight that lack of added benefit to using a contextual retrieval session during this senstive period, as the contextual extinction session alone is successful for attentuating fear in the adolescent age group.

Consistent with our previous work showing that contextual fear memory suppression in adolescent mice is associated with a lack of synaptic potentiation in the basal amygdala (BA)[4], we observed no change in excitatory synaptic transmission in P29 mice after fear conditioning. This is in contrast to fear conditioned adult mice that showed an enhanced excitatory synaptic transmission in the BA (Supplementary Figure 9). While attempts at persistent contextual memory attenuation, but not contextual extinction, caused a depression of excitatory synaptic transmission in the BA of adult mice (Supplementary Figure 9), the excitatory synaptic transmission remained

unaffected in P29 mice irrespective of whether they underwent paradigms for persistent contextual memory attenuation or contextual extinction only and these results are consistent with the persistent attenuation of contextual memory in adolescent mice (Supplementary Figure 10). These data suggest that such persistent attenuation of adult fear memory involves the depotentiation of glutamatergic synapses in the BA while similar attenuation of adolescent fear memory might be independent of BA glutamatergic synapses.

Supplementary References

1 Low, R. J., Gu, Y. & Tank, D. W. Cellular resolution optical access to brain regions in fissures: imaging medial prefrontal cortex and grid cells in entorhinal cortex. *Proceedings of the National Academy of Sciences of the United States of America* **111**, 18739-18744, doi:10.1073/pnas.1421753111 (2014).

2 Monfils, M. H., Cowansage, K. K., Klann, E. & LeDoux, J. E. Extinction-reconsolidation boundaries: key to persistent attenuation of fear memories. *Science* **324**, 951-955, doi:10.1126/science.1167975 (2009).

3 Rao-Ruiz, P. *et al.* Retrieval-specific endocytosis of GluA2-AMPARs underlies adaptive reconsolidation of contextual fear. *Nat Neurosci* **14**, 1302-1308, doi:10.1038/nn.2907 (2011).

4 Pattwell, S. S., Bath, K. G., Casey, B. J., Ninan, I. & Lee, F. S. Selective early-acquired fear memories undergo temporary suppression during adolescence. *Proceedings of the National Academy of Sciences of the United States of America* **108**, 1182-1187, doi:10.1073/pnas.1012975108 (2011).

5 Peters, J., Dieppa-Perea, L. M., Melendez, L. M. & Quirk, G. J. Induction of fear extinction with hippocampal-infralimbic BDNF. *Science* **328**, 1288-1290, doi:10.1126/science.1186909 (2010).

6 Akers, K. G. *et al.* Hippocampal neurogenesis regulates forgetting during adulthood and infancy. *Science* **344**, 598-602, doi:10.1126/science.1248903 (2014).

7 Kim, J. H. & Richardson, R. A developmental dissociation of context and GABA effects on extinguished fear in rats. *Behav Neurosci* **121**, 131-139, doi:10.1037/0735-7044.121.1.131 (2007).

8 Gogolla, N., Caroni, P., Luthi, A. & Herry, C. Perineuronal nets protect fear memories from erasure. *Science* **325**, 1258-1261, doi:10.1126/science.1174146 (2009).

9 Pattwell, S. S. *et al.* Altered fear learning across development in both mouse and human. *Proceedings of the National Academy of Sciences of the United States of America* **109**, 16318-16323, doi:10.1073/pnas.1206834109 (2012).
